# Fixing Flavins: Hijacking a Flavin Transferase for Equipping Flavoproteins with a Covalent Flavin Cofactor

**DOI:** 10.1021/jacs.3c12009

**Published:** 2023-12-04

**Authors:** Yapei Tong, Saniye G. Kaya, Sara Russo, Henriette J. Rozeboom, Hein J. Wijma, Marco W. Fraaije

**Affiliations:** Molecular Enzymology Group, University of Groningen, Nijenborgh 4, 9747 AG Groningen, The Netherlands

## Abstract

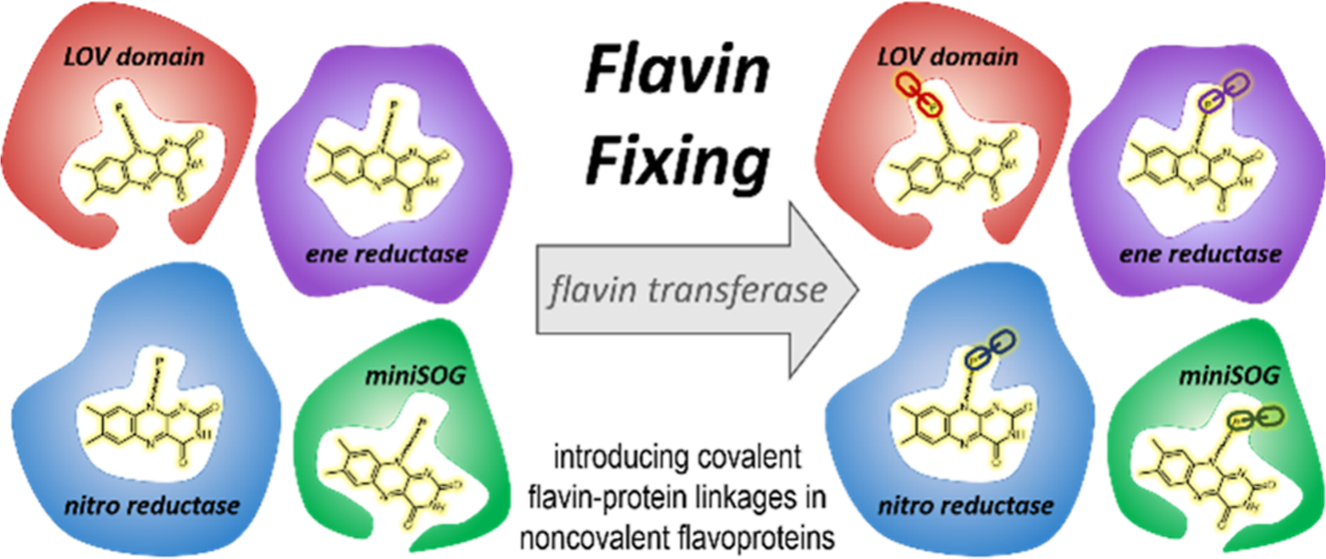

Most flavin-dependent enzymes contain a dissociable flavin cofactor. We present a new approach for installing in vivo a covalent bond between a flavin cofactor and its host protein. By using a flavin transferase and carving a flavinylation motif in target proteins, we demonstrate that “dissociable” flavoproteins can be turned into covalent flavoproteins. Specifically, four different flavin mononucleotide-containing proteins were engineered to undergo covalent flavinylation: a light-oxygen-voltage domain protein, a mini singlet oxygen generator, a nitroreductase, and an old yellow enzyme-type ene reductase. Optimizing the flavinylation motif and expression conditions led to the covalent flavinylation of all four flavoproteins. The engineered covalent flavoproteins retained function and often exhibited improved performance, such as higher thermostability or catalytic performance. The crystal structures of the designed covalent flavoproteins confirmed the designed threonyl-phosphate linkage. The targeted flavoproteins differ in fold and function, indicating that this method of introducing a covalent flavin-protein bond is a powerful new method to create flavoproteins that cannot lose their cofactor, boosting their performance.

## Introduction

Flavoproteins are proteins that use flavin adenine dinucleotide (FAD) or flavin mononucleotide (FMN) as cofactors for their function. Most flavoproteins contain FAD or FMN as a dissociable cofactor. Only a minority of flavoproteins contain a covalently attached flavin cofactor, mostly involving a FAD.^1^ So far, nine different types of covalent flavin-protein linkages have been identified in natural flavoproteins. The roles and mechanisms of the different modes of covalent flavinylation have been studied in detail.^2,3^ It has been shown that the covalent bond between the flavin and the host protein can result in a more stable protein structure,^4−6^ increased enzyme activity,^6,7^ tuned redox properties of the enzyme,^7,8^ prevent dissociation of the cofactor,^9^ and facilitate electron transfer.^10^ Most of the flavin-protein linkages involve a covalent bond between the benzyl moiety of the redox-active isoalloxazine ring system of FMN or FAD. Such a covalent linkage can be formed by a self-catalytic process. Only in one case is there tethering via the phosphate group of FMN. This covalent linkage was found to be formed with the help of an extracytosolic bacterial flavin transferase, which uses FAD for attaching the FMN part to the target threonine or serine. Genome sequence analysis has revealed that such covalently flavinylated proteins are common in bacteria and seem to be involved in various extracellular redox processes, often facilitating the transfer of single electrons.

We have recently shown that the above-mentioned flavin transferase can be used for directed labeling of target proteins with a covalent flavin, a flavin analogue, or even a nicotinamide group. By equipping a protein with an N- or C-terminal peptide that includes a flavinylation recognition site (DxxxGA[**T**/**S**], flavinylated amino acid in bold), FMN can be enzymatically attached using a truncated, and therefore soluble, version of the bacterial flavin transferase from *Vibrio cholerae* (ApbE).^11−13^ We termed this approach Flavin-tagging and have successfully applied it for labeling several proteins with FMN or FMN derivatives with altered chromogenic, fluorescence, and redox properties.^13,14^ The Flavin-tag system can be used as a convenient tool for decorating a target protein with a flavin-based probe. Selective covalent labeling can be achieved in vivo (by coexpressing the target protein and the flavin transferase) and in vitro (by incubating the target protein with the flavin transferase and FAD).

Based on the Flavin-tag system, we set out to explore whether the flavin transferase can also be exploited to install a catalytically active flavin in a covalent manner into a target flavoprotein. To investigate this, we targeted four FMN-containing proteins that differ in structure and function and for which structural and functional properties are known: the light-oxygen-voltage (LOV) domain protein from *Pseudomonas putida* KT2440 (PpSB1-LOV), the mini singlet oxygen generator engineered from *Arabidopsis thaliana* phototropin 2 (miniSOG), a bacterial nitroreductase (BtNR), and an old yellow enzyme (OYE)-type ene reductase from *Thermus scotoductus*.

LOV domain proteins contain a noncovalently bound FMN and belong to the Per-Arnt-Sim domain superfamily. They have been developed into flavin-based fluorescent proteins for cell imaging purposes. Due to their unique properties such as fast folding, efficient fluorophore incorporation, and oxygen-independent maturation, they often outperform alternative protein-based reporters, such as green fluorescent protein and its derivatives.^15−17^ Still, LOV-based protein reporters are the target of protein engineering studies in order to improve some of their properties. Many efforts have been made to improve their brightness, thermal stability, and pH tolerance.^18−20^

MiniSOG proteins have recently been engineered, starting from LOV proteins. The miniSOG that we studied has been engineered by truncating phototropin 2 from *A. thaliana* and introducing several mutations that eliminate the ability to form, upon light exposure, a reversible flavin-cysteine covalent adduct.^21^ As a result, this small protein (106 residues) catalyzes the light-powered production of singlet oxygen. MiniSOG was shown to be a valuable tool in cell biology where it can be used as a photosensitizer.

As a third flavoprotein prototype, we selected an FMN-containing nitroreductase. Nitroreductases are considered valuable biotechnological tools. They are intensely studied as prodrug-activating enzymes acting on nitro-containing compounds, enabling the treatment of cancer and infections.^22^ They are also considered to be valuable biocatalysts for producing amines.^23^ For our work, we focused on a newly discovered nitroreductase from *Bacillus tequilensis* (BtNR).

To extend the approach to another class of enzymes that are highly relevant for biocatalytic applications, we included an ene reductase for the OYE family:^24^ the thermostable OYE from *T. scotoductus*.^25^

As mentioned above, the covalent attachment of a flavin cofactor can bring advantages to a flavoprotein.^4−6^ Yet, all known representatives of the above-mentioned flavoproteins contain a dissociable FMN cofactor. Hence, we envisioned that the covalent tethering of FMN could introduce beneficial effects, such as eliminating the risk of dissociation of the cofactor and improving the stability. Opposite to the most common naturally occurring flavin-protein linkages, we aimed at covalent attachment via the phosphate moiety of FMN. By this, we did not anticipate a detrimental effect on the intrinsic chromogenic, fluorescence, and catalytic properties of the flavin cofactor as these properties reside in the isoalloxazine ring system, which is relatively far from the phosphate moiety. Based on structure-based modeling, we introduced recognition sites for the truncated flavin transferase in all four targeted flavoproteins. For each targeted enzyme, two or three alternative attachment sites were introduced. For introducing the flavinylation sites, 5–7 amino acids had to be replaced in each engineered variant. Upon coexpression with the flavin transferase, all ten redesigned mutants were purified and characterized. All variants were found to contain covalent FMN and to be functional, revealing that the flavin transferase can be used to covalently tether a flavin cofactor to an otherwise noncovalent flavoprotein. Such covalent variants are highly attractive for applications as tethering the cofactor can boost the performance of flavoproteins, as is shown for the generated covalent variants in this study.

## Results and Discussion

### Computationally Aided Design of Flavin Attachment Sites

**Figure 1:**
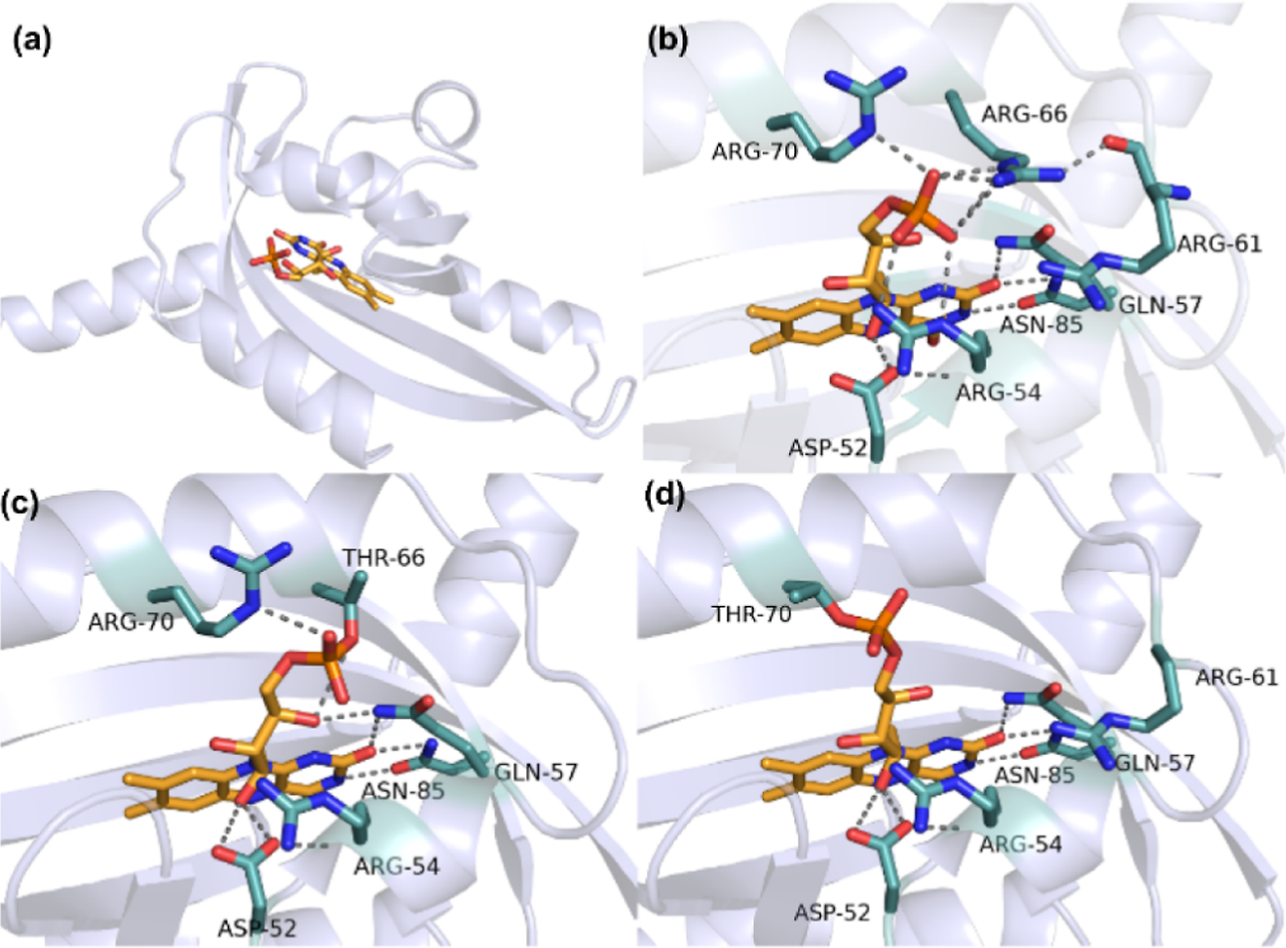
FMN binding pocket of PpSB1-LOV and the modeled variants. (a) Overall structure of the wild-type PpSB1-LOV protein (PDB: 5J3W). (b) Close-up view of the FMN binding state of PpSB1-LOV wild-type. (c) Predicted FMN binding state of PpSB1-LOV-F1. (d) Predicted FMN binding state of PpSB1-LOV-F2.

It was previously established that the flavin transferase ApbE from *V. cholerae* recognizes a specific sequence motif for the covalent attachment of FMN to a threonine or serine.^11,13^ This motif is flanked by a fully conserved aspartate at its N-terminus, which is essential for the covalent flavinylation by ApbE. The aspartate is separated by five (semi)conserved amino acids from the flavin-linkage residue, a threonine, or serine. This makes a motif of seven residues: D-[GAIQ]-[IALVF]-[ST]-G-A-[ST]. Starting from the crystal structure 5J3W,^26^ two potential anchor points near the phosphate moiety of the bound FMN were identified by visual inspection: Arg66 and Arg70. Only threonine was considered an anchoring residue as this residue resulted in a higher flavinylation efficiency in Flavin-tags compared with that of serine.^13^ The six preceding residues of the motif were subsequently chosen by established rational protein engineering considerations such as to minimize the structural change at a position, e.g., retaining a glycine at position 65 when compatible with the motif, mutating to an alanine whenever in doubt (e.g., D62A, not D62ILVF), and minimizing hydrophobic exposure at the surface (e.g., Q63S, not Q63T, which would introduce a water-exposed hydrophobic methyl group).^27^ This resulted in two PpSB1-LOV variants, each with six point mutations, which are further referred to as PpSB1-LOV-F1 (D60D + R61A + D62A + Q63S + L64G + G65A + R66T) and PpSB1-LOV-F2 (L64D + G65G + R66A + A67S + R68G + I69A + R70T) (Figure 1).

Starting with the thus-modeled 3D structures of the two mutants, MD simulations were carried out to evaluate whether the newly introduced mutations and covalent flavin-protein bonds would be compatible with the existing structure and function. For the MD simulations, an established protocol was used, which has been successfully used to screen mutants for improved (thermo)stability.^28,29^ The protocol includes five independent MD simulations for each variant, to obtain better conformational sampling compared with a single MD simulation,^30,31^ and includes visual inspection of the five averaged trajectories. The simulations suggested only some increase in backbone flexibility for both mutants, but these were so limited that it appeared likely that the proteins would still form stable structures. The MD simulations also indicated that for both mutants, the isoalloxazine ring of the FMN stays bound in the active site without significant differences with the wild-type protein. Furthermore, AlphaFold modeling was used as an independent check for the structural viability of the two designed PpSB1-LOV variants.^32^ The resulting models did not reveal significant changes in the backbone structure as compared with that of the wild-type protein. Therefore, both variants were selected for experimental characterization.

### Expression and Purification of the PpSB1-LOV Variants in the Absence of the Flavin Transferase

To study whether the two designed PpSB1-LOV mutants can still be expressed, fold, and bind FMN, we first expressed wild-type PpSB1-LOV and the two PpSB1-LOV mutants (F1 and F2) without ApbE using *Escherichia coli* BL21-AI as the expression host and purified the respective proteins. Expression levels were high and similar for all proteins (about 300 mg per liter of culture). Both mutant proteins could be purified by affinity chromatography and were called PpSB1-LOV-F1a and -F2a, respectively. While PpSB1-LOV-F1a was purified as a yellow protein, indicative of bound FMN, the other variant was totally colorless. This indicates that PpSB1-LOV-F2a had lost its capacity to bind FMN in a tight manner (Figure S1). Similar to wild-type PpSB1-LOV, purified PpSB1-LOV-F1a was found to be sensitive to blue light (Figure S2a). The absorbance spectrum of PpSB1-LOV-F1a in the dark state displayed the typical features of an oxidized flavin spectrum, with two major absorbance maxima at 370 and 450 nm. Upon exposure to blue light, the color bleached, and the formed absorbance spectrum revealed a less defined spectrum. In the dark, the oxidized spectrum was completely restored in 2 h (Figure S2b).

After purifying the apo form of PpSB1-LOV-F2a, we incubated the protein with ApbE and FAD. However, this attempt to perform in vitro covalent flavinylation failed. No covalent FMN was incorporated as judged from detecting no fluorescence upon SDS-PAGE. The failure to covalently incorporate FMN by the flavin transferase is probably due to the fact that in the fully folded state, the flavinylation recognition motif is not accessible for the transferase.

PpSB1-LOV-F1, when expressed in the absence of the flavin transferase, still retained the ability to bind FMN as a dissociable cofactor, whereas PpSB1-LOV-F2 had lost affinity for FMN as it was purified without any flavin binding (Figure S1). The difference in cofactor affinity can be explained by analyzing the modeled structures. The binding of FMN to proteins relies for a large part on the interactions with the phosphate group of the cofactor. In the studied LOV protein, the phosphate moiety of FMN is relatively exposed to the solvent and seems to be bound through electrostatic interactions with three arginines in wild-type PpSB1-LOV: R54, R66, and R70 (Figure 1b).^26^ PpSB1-LOV-F1 has lost only one of these arginines (R66), while PpSB1-LOV-F2 lacks two arginines (R66 and R70) as a result of introducing the flavinylation recognition sequence (Figure 1c,d). This difference can explain that PpSB1-LOV-F1 can still be produced as a holoprotein in the absence of the flavin transferase.

### Coexpression of PpSB1-LOV Variants with the Flavin Transferase

**Figure 2:**
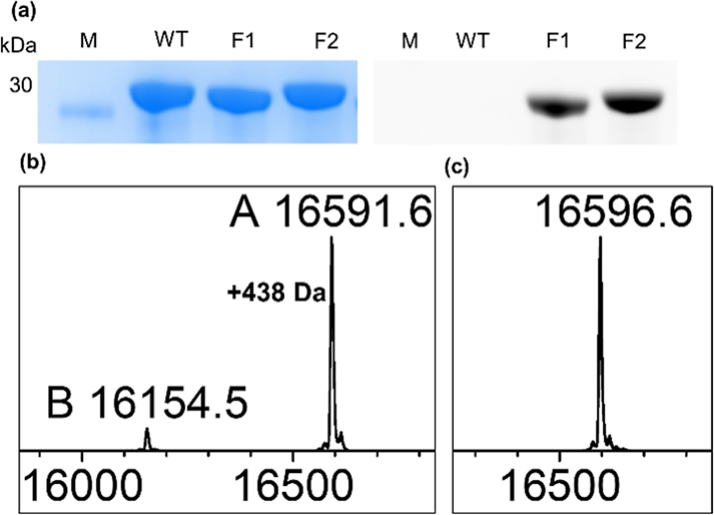
Expression and mass identification of PpSB1-LOV and its variants. (a) Polyacrylamide gel (SDS-PAGE) of purified PpSB1-LOV proteins: wild-type, variant F1, and variant F2. In-gel fluorescence (right) and protein staining (left) of the same gel are shown. (b) Electrospray ionization mass spectral analysis of PpSB1-LOV-F1. (c) Electrospray ionization mass spectral analysis of PpSB1-LOV-F2. Cells were grown at 30 °C for 20 h in 30 mL TB.

To probe whether FMN can be covalently attached to the two PpSB1-LOV variants during folding, we coexpressed the redesigned proteins with the flavin transferase ApbE. Except for the flavin transferase, the FAD synthase from *Corynebacterium ammoniagenes* (CaFADS) was also coexpressed to increase the intracellular levels of FAD. Previous work has shown that coexpression of both enzymes boosts the levels of flavin incorporation into the Flavin-tag.^14^ For the coexpression experiments, the plasmid pRSF-Duet1 (expressing ApbE and CaFADS) was cotransformed with the plasmid carrying a PpSB1-LOV-encoding gene. As a reference, wild-type PpSB1-LOV was also coexpressed with the flavinylation machinery and purified. Intriguingly, all expressed PpSB1-LOV proteins could be purified as bright yellow proteins. Expression levels were high and similar for all proteins (about 300 mg per liter of culture). The proteins were analyzed via SDS-PAGE and subsequent in-gel fluorescence detection (for detecting covalent flavin), followed by protein staining. This revealed that for both designed LOV variants, clear in-gel fluorescence was observed, indicating that FMN was successfully covalently incorporated into the proteins (Figures 2a and S3). As expected, the wild-type protein did not yield in-gel fluorescence as it contains a dissociable FMN, which dissociates upon SDS treatment. To further verify that PpSB1-LOV-F1 and -F2 were truly covalently flavinylated by FMN, protein samples were subjected to mass spectrometry analysis. As expected, the covalent attachment of FMN (manifested as a +438 Da mass gain) on both LOV proteins was readily detected by ESI-MS (Figure 2b,c).

Except for the mass of PpSB1-LOV-F1 conjugated with FMN, a small fraction of protein without modification was detected. To determine whether part of the protein contains noncovalently bound FMN, similar to the wild-type protein, a sample of PpSB1-LOV-F1 was denatured and precipitated with TCA.^33^ After centrifugation, only a minor amount of FMN was detected corresponding to 1–2% of dissociable FMN. For PpSB1-LOV-F2, only a species of mass corresponding to the protein containing a covalently tethered FMN was detected. As expected, the wild-type protein resulted in only a mass species with the predicted mass of the protein. The data clearly show that ApbE, during coexpression, can insert a covalent FMN into the two redesigned LOV proteins.

### Optimization of Covalent Flavinylation of PpSB1-LOV Variants

It was found that fully flavinylated PpSB1-LOV-F2 could be easily obtained with 100 mg/L riboflavin when expressed at 24 or 30 °C. Yet, for variant F1, MS analysis revealed that part of the protein did not contain covalent FMN (Figure 2b). Previous studies showed that culturing conditions (temperature and riboflavin concentration) could influence the flavinylation of the target protein containing a Flavin-tag.^14^ Therefore, we optimized the induction temperature and riboflavin concentration to boost the incorporation of covalent FMN into PpSB1-LOV F1. The highest level of covalent flavinylation of PpSB1-LOV-F1 (91%) was obtained using a temperature of 30 °C for expression and adding 150 mg/L riboflavin to the medium (Figure S4).

### Effects of Covalent Incorporation of FMN on the Photochemical Properties of PpSB1-LOV

**Figure 3:**
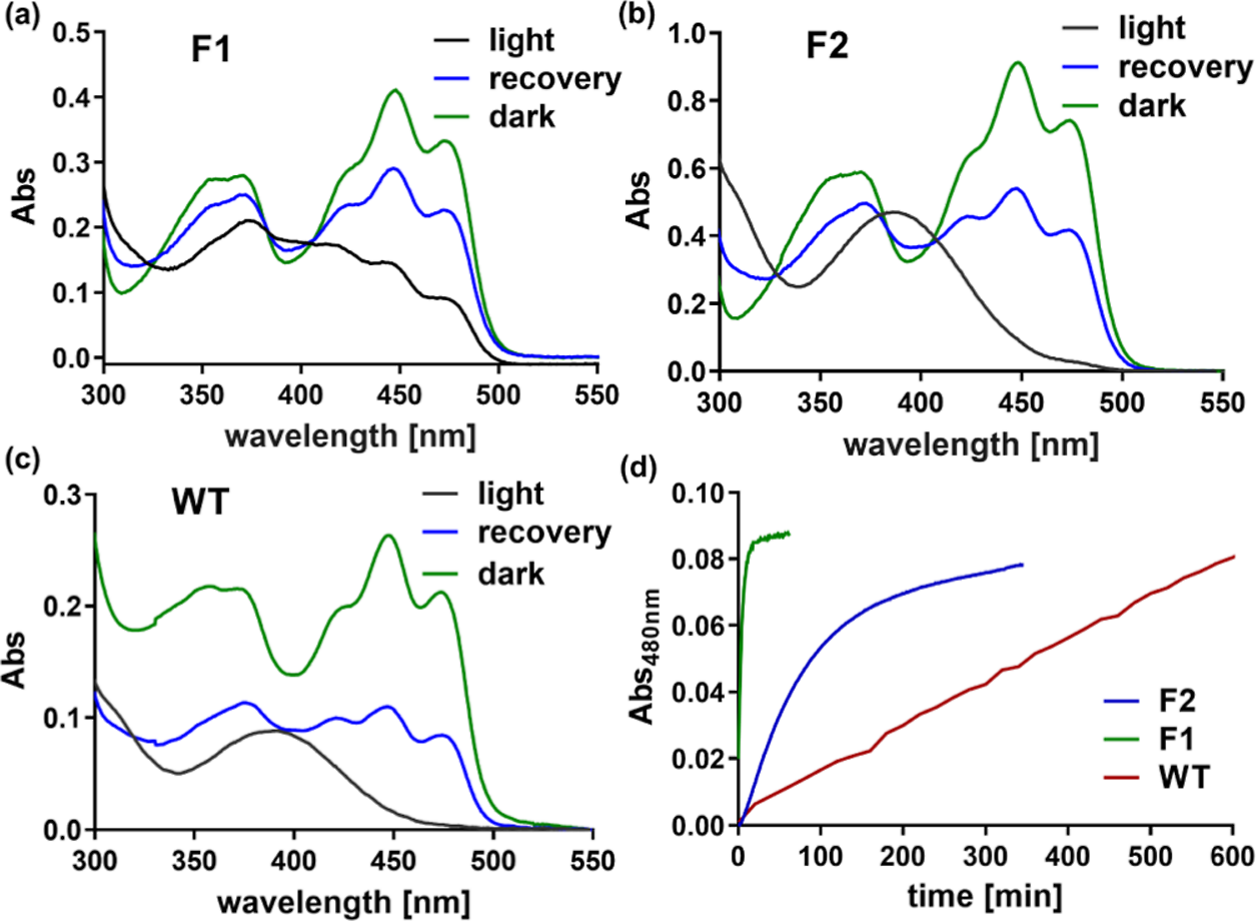
UV/vis spectra for PpSB1-LOV variants. Spectra of (a) PpSB1-LOV-F1, (b) PpSB1-LOV-F2, and (c) wild-type PpSB1-LOV. The dark-adapted (black) and dark-recovered states (green) are shown, while a spectrum during the recovery process is also shown (blue). (d) Recovery traces of wild-type PpSB1-LOV and the two covalent variants (F1 and F2) were recorded as absorbances at 480 nm. Spectra were recorded at 25 °C in the dark after 30 s of blue-light illumination: wild type (red), F1 (green), and F2 (blue).

Next, we investigated the blue-light response of the two variants. We observed that, compared with the wild-type LOV protein, PpSB1-LOV-F1 and -F2 undergo the same photochemical cycle as observed by monitoring the flavin absorbance spectra before and after light exposure (Figure 3a–c). Clearly, the covalent anchoring of FMN does not prevent the photochemistry of the flavin cofactor typical for LOV proteins. As both engineered variants were found to contain covalent FMN and showed to be sensitive to blue light, both variants were studied in more detail.

We measured the absorbance spectra of all three PpSB1-LOV variants (wild type, F1, and F2) to investigate whether there are any changes upon covalent tethering of the flavin cofactor. Both F1 and F2 display highly similar absorbance spectra when compared with those of the wild-type protein with absorption and fluorescence emission maxima of 447 and 490 nm, respectively (Figure S5a). Except for showing absorbance maxima at the same wavelengths, the fine structures of the spectra were also virtually identical.

The photochemical reactions of LOV domain proteins that occur between the C4a atom of FMN and a strictly conserved cysteine in the LOV domain are induced by blue light illumination (Figure S5b).^34−36^ The photoinduced reaction was not affected by the altered mode of cofactor binding as both redesigned LOV proteins could be swiftly converted into the dark state after irradiation by blue light (Figure 3d). Interestingly, the dark recovery was found to be relatively fast for the variants that contain a covalent FMN. We determined the rate of dark recovery (τ_rec_) for all FMN-containing PpSB1-LOV proteins: wild-type, F1a with noncovalent FMN, F1 with covalent FMN, and F2 with covalent FMN. Both variants carrying covalent FMN displayed a relatively short recovery lifetime (τ_rec_) when compared with that of the wild-type protein. Variant F1 displays the fastest dark recovery, accelerating from almost 1 day (1150 min) for wild-type PpSB1-LOV to about 5 min in the case of PpSB1-LOV-F1 (Table 1). The F1 variant that could be obtained with a noncovalently bound FMN (F1a) also recovered relatively fast but somewhat slower when compared with the same protein that has a covalent protein-FMN linkage (Table 1 and Figure S2). It indicates that the acceleration in dark recovery is not only due to the introduced covalent flavin-protein bond. The dark recovery of the PpSB1-LOV-F2 variant was somewhat slower than that of PpSB1-LOV-F1 but still significantly faster than that of the wild-type protein (49 vs 1150 min).

**Table 1:** Characterization of the Studied Flavoproteins^a^

LOV protein variant	covalent FMN incorporation (%)	dark recovery (τ_rec_) (min)	fluorescence quantum yield (Φ)	*T*_m_ (°C)
WT	0	1150 ± 430	0.10 ± 0.01	54.0
F1a	0	23.0 ± 6.6	0.01 ± 0.001	56.5
F1	91	4.6 ± 0.5	0.35 ± 0.02	64.5
F2	100	49.1 ± 7.1	0.20 ± 0.01	57.5

miniSOG variant	covalent FMN incorporation (%)	singlet oxygen production (%)	*T*_m_ (°C)
WT	0	100	66
F1	100	150	56
F2	100	202	61

BtNR variant	covalent FMN incorporation (%)	*k*_cat_ (s^–1^)	*K*_M_ (μM)	*k*_cat_/*K*_M_ (s^–1^ mM^–1^)	*T*_m_ (°C)
WT	0	32 ± 2.3	64 ± 11.5	500	58
F1	90	10 ± 2	31 ± 18	320	53
F2	90	23.4 ± 2	59.5 ± 16.3	390	63
F3	100	4.5 ± 0.2	42 ± 6.6	110	51

OYE variant	covalent FMN incorporation (%)	*k*_obs_ (s^–1^)	*T*_m_ (°C)
WT	0	6.2 ± 0.1	94
F1	100	8.0 ± 0.9	79
F2	100	7.1 ± 1.4	59
F3	100	8.1 ± 0.3	60

a All proteins were coexpressed with ApbE, except for PpSB1-LOV-F1a, which was expressed without ApbE coexpression. All data were derived from three independent measurements.

In addition to characterizing the photocycle, all variants were also characterized concerning other features: thermostability, fluorescence quantum yield, and pH stability. Fluorescence quantum yields (Φ) were determined using fluorescein as a reference (Φ = 0.91).^37^ In order to determine the Φ values, which were evaluated at the fully fluorescent on-state brightness condition of all proteins, the proteins were kept in the dark prior to the measurement. Interestingly, PpSB1-LOV-F1 and -F2, both containing covalent FMN, exhibited a quantum yield 3.5 times and 2 times higher than that of the wild-type LOV protein, respectively (Table 1). The version of PpSB1-LOV-F1 that contained noncovalent FMN (PpSB1-LOV-F1a) exhibited a ten times lower fluorescence quantum yield compared to that of the wild type. These data show that the covalent attachment of the FMN cofactor results in brighter fluorescent LOV proteins.

We explored whether there are other benefits of the covalent attachment of FMN. We found that the F1 and F2 variants containing covalent FMN significantly outperformed the wild-type protein in terms of thermostability. The effect was largest for PpSB1-LOV-F1, which displayed an apparent melting temperature (*T*_m_) that was 10.5 °C higher when compared with that of the wild-type LOV protein (Table 1). Analysis of PpSB1-LOV-F1 that contained dissociable FMN revealed that the covalent linkage contributes significantly to higher thermostability as this variant only had a 2.5 °C higher apparent melting temperature (Table 1).

### Crystal Structure of PpSB1-LOV-F1

**Figure 4:**
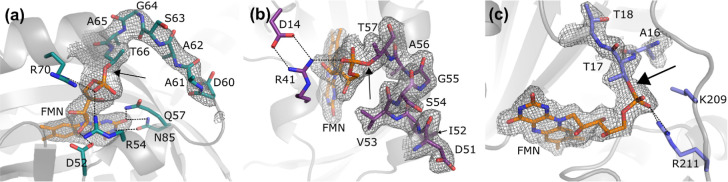
Crystal structures of the engineered covalent flavoproteins. (a) PpSB1-LOV-F1, (b) miniSOG-F2, and (c) BtNR-F3. The mutated residues, shown in the stick presentation, are colored green, purple, and slate, respectively. FMN atoms, in sticks, are colored orange. 2Fo-Fc electron density maps, in gray, of the mutated residues and FMN are contoured at 1.0 σ. Hydrogen bonds are shown as dashed lines, and large arrows indicate the bond linking the phosphate to the threonine residue.

To gain insight into the structural consequences of the covalent tethering and the introduced mutations, we determined the crystal structure of PpSB1-LOV-F1. The dimeric structure of PpSB1-LOV-F1 to 2.4 Å was solved by molecular replacement using the wild-type PpSB1-LOV structure (PDB code 3SW1).^38^ Superimposing the crystal structure of wild-type PpSB1-LOV and the covalent variant, PpSB1-LOV-F1, reveals that the two structures only deviate slightly (rmsd on 122 Cα atoms: 1.3 Å). The 6 mutations in PpSB1-LOV-F1 have no influence on the overall structure. Residues 61–63 are in a loop, and residues 64–66 are part of an α-helix similar to other LOV domain proteins. The isoalloxazine and ribityl moieties of FMN have the same interactions with the protein side chains. The phosphate atom, however, has a different position, 1.9 Å away toward Thr66, to which it is covalently attached (Figure 4a), by a changed angle of C4′–C5′–O5′. From the interactions with guanidino groups of Arg54, Arg61, Arg66, and Arg77 in wild-type PpSB1-LOV, only Arg54 and Arg70 are retained due to the mutations.

A comparison of the modeled structure of PpSB1-LOV-F1 (vide supra) and its crystal structure reveals an rmsd of 1.0 Å (Figures 1c and 4a). The isoalloxazine ring resides at the same position in both structures. The most significant deviations are observed in the N- and C-terminal regions and around the threonyl-flavin linkage. This includes a shift of the α-helix containing residues 64–76 (containing Thr66) of 1.4 Å toward its C-terminus. The conformation of residues 59–65 is also quite different in the crystal structure, mainly through the shift of the α-helix and the backbone angles of Gly64. The predicted structure has a hydrogen bond between O3 of the phosphate and NH_2_ of Arg70, which is not observed in the crystal structure. The rotamer conformation of Thr66 is correctly predicted.

### Covalent Flavinylation of the Photosensitizing miniSOG

To investigate whether the Flavin-fixing approach also works for other flavoproteins, we selected a miniSOG that has been engineered from phototropin 2 from *A. thaliana*. MiniSOGs have a similar structure when compared with that of LOV domain proteins and also contain a dissociable FMN. It has been shown that dissociation of the flavin cofactor from miniSOG can cause drastic effects on its photosensitizing abilities. Again, inspection of the crystal structure of this target flavoprotein revealed two potential sites for covalent attachment (V53 and R57). Except for introducing a threonine residue at the respective positions, analogous to the engineering of PpSB1-LOV, the 6 residues before each site were also mutated to carve a recognition site for the flavin transferase into miniSOG as was done for the LOV protein (47-DAASGAT-53 for miniSOG-F1 and 51-DIVSGAT-57 for miniSOG-F2, respectively). Expression and subsequent purification of the two engineered miniSOG variants, coexpressed with the flavin transferase, resulted in yellow-colored proteins. SDS-PAGE and MS analyses revealed that both proteins were fully covalently flavinylated (Figure S6). The proteins were also tested for their ability to generate singlet oxygen upon light exposure. Gratifyingly, the covalent variants were found to be competent photosensitizers. In fact, both covalent variants were more effective in generating singlet oxygen (Table 1, Figure S7). While the two covalent variants retained their function, the covalent coupling led to a slight decrease in thermostability (Table 1).

To analyze the effects of covalent flavinylation on the structure of miniSOG, miniSOG-F2 was crystallized. The structure of miniSOG-F2 was determined with molecular replacement to 2.0 Å resolution using the model of miniSOG (PDB code 6GPU).^39^ The asymmetric unit contains two miniSOG-F2 domains. The packing of these molecules and two symmetry-related domains is quite peculiar. According to the PISA Web server, the assembly contains an A2B2, tetramer formation. As observed for the LOV domain protein variants (vide supra), the structures of miniSOG-F2 and miniSOG are very similar (rms deviation on Cα atoms of 0.55 Å). The mutated residues of 51ATVQKIR57 to 51DIVSGAT57 are clearly visible in electron density (Figure 4b). The Cα atoms in the α-helix containing residues 50–63 deviate by 1.5 Å at Gly55 (at most) and 0.3 Å at Thr57. All backbone dihedral angles φ and ψ of the helix are within the energetically favored regions. Electron density clearly confirms the introduced phosphoester bond between OG1 of Thr57 and the phosphate of FMN. The isoalloxazine group remains embedded at the same position in the protein. Also, the ribityl moiety has a conformation similar to that in the homologous proteins. Only the angle of C5′–O5′–P is different and is bending toward Thr57. The phosphate group in miniSOG-F2 is further stabilized by the guanidino group of Arg41. This protein residue is fixed by a salt bridge with Asp14. The interaction in miniSOG with Arg57 is not observed, as in miniSOG-F2, it has been mutated to Thr57. No other contacts are lost or gained by the mutated residues. Overall, the introduction of the flavin-protein linkage has a minimal impact on the structure and function of miniSOG.

### Covalent Flavinylation of the Nitroreductase BtNR

The Flavin-fixing method was also applied to another FMN-containing enzyme that is structurally unrelated to the LOV domain proteins. We recently discovered a novel BtNR that is capable of reducing a variety of nitro compounds. Such reductases are considered interesting biocatalysts for the production of amines and are also considered for use as prodrug-activating enzymes in cancer therapy. Inspection of the crystal structure of BtNR revealed three potential sites for flavinylation: three neighboring residues that are positioned close to the FMN phosphate (R15, H16, and A17). Three corresponding mutants were prepared that involved introducing flavinylation recognition sites of 7 residues: BtNR-F1, BTNR-F2, and BtNR-F3. All three redesigned proteins could be expressed and purified as yellow proteins. SDS-PAGE and MS analyses revealed that (Figure S8), in contrast with wild-type BtNR, all mutant proteins contained covalent FMN. Yet, the degree of covalent incorporation varied from 90 to 100% (Table 1). All three variants were found to be active as nitroreductase as they showed activity toward substrate nitrofurazone (Table 1 and Figure S9). All these BtNR variants did not differ significantly from the WT in *K*_m_, *k*_cat_, or *k*_cat_/*K*_m_ values. Thermostabilities of the three covalent NRs varied. While BtNR-F1 and BtNR-F3 displayed lower stabilities (*T*_m_ lowered by 5–7 °C), BtNR-F2 was found to be more thermostable when compared with wild-type BtNR (*T*_m_ increased by 5 °C) (Table 1). The results show that the position for introducing a threonine to covalently coupled FMN is somewhat flexible as all engineered NRs could be converted into a covalent flavoprotein.

The dimeric BtNR-F3 structure was determined to be 2.0 Å. Rms deviation with the structure of BtNR wt is 0.5 Å. The mutated residues from BtNR wt 11AYNFRHA17 to BtNR-F3 11DGLSGAT17 represent the flavin fix site. Intriguingly, for the first 15 residues of both chains, including Asp11, no electron density was observed, and these residues are probably flexible. Electron density is well defined from Ala16 onward up to the C-terminus. In BtNR wt, residues 1–14 reside in an α-helix. The FMN phosphate group (Figure 4c) shifts 1.6 Å toward OG1 of Thr17, to which it is covalently attached. This linkage causes strain in nearby protein residues. Despite the Cα atom of Thr17 shifting by 2.7 Å compared to that of wt Ala17, the covalent bond is formed. The phosphate group in BtNR wt is stabilized by the side chains of Arg15, Lys209, and Arg211 and the backbone nitrogen of Ala17. In BtNR-F3, besides the covalent linkage to Thr17, the interaction with R211 remains.

### Covalent Flavinylation of an OYE-Type Ene Reductase

To probe whether the Flavin-fixing approach could also be used for yet another type of flavoenzyme, we selected an OYE-type ene reductase. Such reductases are highly valued biocatalysts for preparing fine chemicals. Analogous to the other targeted flavoenzymes, we expressed three variants of the thermostable OYE from *T. scotoductus*. Gratifyingly, all three 7-fold mutant proteins were found to be fully covalently flavinylated (Figure S10) and retained activity as ene reductases (Table 1).

## Conclusions

In this work, we present a new and subtle bioorthogonal protein engineering approach (which we coined Flavin-fixing) to switch the cofactor-binding mode from noncovalent to covalent in an FMN-dependent protein. To achieve this, structure-based modeling was performed to carve a sequence recognition motif into the protein structure that would be compatible with the formation of a covalent threonyl-flavin linkage. The motif was based on previous work that has revealed the sequence requirements for a bacterial flavin transferase to use FAD as a substrate to covalently incorporate the FMN part.^11,13^ While it had been shown that such a sequence could be used as an N- or C-terminal tag for labeling target proteins with FMN or other flavin derivatives (the Flavin-tag method),^13^ this study provides evidence that covalent FMN incorporation can also be achieved in flavoproteins that normally harbor a noncovalently bound FMN. While the utilized flavin transferase is normally involved in covalent flavinylation of extracellular bacterial proteins, our study shows that it can be exploited for covalent flavin attachment to various structurally unrelated flavoproteins. The flavinylated proteins remain functional and often display improved properties, such as thermostability and fluorescence quantum yield. The elucidated crystal structures confirm that the covalent attachment does not significantly perturb the binding and microenvironment of the redox active part of the flavin cofactor. The results indicate that the method is amenable to many FMN-containing flavoproteins, as long as a threonine or serine and an accompanying sequence motif can be introduced at a position structurally compatible with the formation of a covalent threonyl/serinyl-phosphate linkage. Clearly, Flavin fixing is not limited to one particular protein fold. We have shown that both α/β fold (LOV proteins) and α + β fold proteins (nitroreductases and ene reductases) can be equipped with a covalent flavin cofactor.

Installing the covalently attached FMN in the target proteins was successful only when expressing the engineered target protein together with the flavin transferase, ApbE. We have also observed that covalent flavinylation could not be achieved when using a folded apo protein (apo PpSB1-LOV-F2) in combination with the flavin transferase. This indicates that covalent incorporation is established cotranslationally during the protein synthesis and folding process. This is in contrast to the mechanism of covalent flavinylation of most natural covalent flavoproteins, in which a covalent bond is formed via the isoalloxazine moiety. For such flavoproteins, it has been observed that covalent flavinylation can also be performed by starting from a folded apoprotein. In that case, a covalent bond is formed by a self-catalytic process. Covalent attachment of a flavin cofactor has previously also been accomplished by using chemically modified, reactive flavin derivatives.^40,41^ Such approaches typically depend on in vitro modification of the target protein and often result in perturbation of the structure and/or function of the respective flavoprotein. Also, the synthesis of such functionalized flavins is typically very cumbersome. Clearly, the herein presented method of covalent anchoring of a flavin cofactor is very attractive. The method merely requires coexpression of the target redesigned protein with a flavin transferase and requires no special additives. The introduction of the flavinylation recognition site has to be carefully designed, for which structural information is essential. Equipping noncovalent flavoproteins with a covalent FMN cofactor is very attractive to improve their performance. For example, it is known that flavin dissociation hampers biotechnological applications as it eliminates enzyme activity and can trigger protein unfolding.^42^ Except for establishing a covalent FMN-protein bond in natural noncovalent flavoproteins, the Flavin-fixing approach can also be used to install alternative flavin cofactors in target proteins. As we have previously shown that the flavin transferase also accepts flavin derivatives, it will also allow the covalent incorporation of unnatural flavins into target proteins. Such a cofactor engineering approach may tune or even introduce new functionalities into proteins.

## Data Availability

Atomic coordinates and structure factors for the reported crystal structures have been deposited with the Protein Data Bank (PDB), with the following accession numbers: 8Q5E for PpSB1-LOV-F1, 8Q5F for miniSOG-F2, and 8Q5G for BtNR-F3. The structural data used in this study are available in the PDB database under accession codes: 3SW1 and 5J3W (wild-type PpSB1), 6GPU (miniSoG), and 8QYG (wild-type BtNR). The experimental data generated in this study are provided in the Supporting Information. All data and materials supporting the findings in the manuscript are available from the corresponding author upon reasonable request.

## Supporting Information Available

Materials and methods; instrumentations; protein sequences; UPLC-ESIMS data; absorption spectra of purified PpSB1-LOV-F2; photochemical property of PpSB1-LOV-F1a; full images for gel data; effect of riboflavin concentration and temperature on covalent flavinylation of PpSB1-LOV-F1; spectral properties and photocycle of the PpSB1-LOV protein; expression and mass identification of mini-SOG and its variants; detection of singlet oxygen generated by the miniSOG variants; expression and mass identification of BtNR and its variants; steady-state kinetics of BtNR wild-type and flavinylated mutants F1-3; expression and mass identification of OYE (3HGJ) and its variants; all the primers used for cloning BtNR variants in this study; sequences of proteins used in this study; molar extinction coefficients of PpSB1-LOV, miniSOG, and BtNR proteins; and data collection and refinement statistics (PDF)
