# Supplementary material for: Fixing Flavins: Hijacking a Flavin Transferase for Equipping Flavoproteins with a Covalent Flavin Cofactor
Source: J Am Chem Soc. 2023 Dec 4;145(49):27140–8. doi: 10.1021/jacs.3c12009 (PMC10722498; doi:10.1021/jacs.3c12009)
Supplement: Supplementary file 1 — ja3c12009_si_001.pdf [file ja3c12009_si_001.pdf]

Supplementary Information

Fixing flavins: hijacking a flavin transferase for equipping flavoproteins with a covalent flavin cofactor

Yapei Tong, Saniye G. Kaya, Sara Russo, Henriette J. Rozeboom, Hein J. Wijma, Marco W. Fraaije*

* Corresponding author: Marco W. Fraaije, m.w.fraaije@rug.nl

Molecular Enzymology group, University of Groningen, Nijenborgh 4, 9747AG, Groningen, The Netherlands

Contents

Experimental Section 3

Materials 3

Computational modelling of PpSB1-LOV variants 3

Plasmid construction 4

Protein purification and characterization 4

Determining non-covalently bound FMN 5

Thermal stability determination 6

ESI-MS analysis of modified proteins 6

Fluorescence, photobleaching and photorecovery measurements 6

Singlet oxygen detection 7

Enzyme kinetics of BtNR 7

OYE activity measurement 8

Crystallization and structural determination 8

Supplementary Figures and Tables 10

Figure S1 10

Figure S2 11

Figure S3 12

Figure S4 13

Figure S5 14

Figure S6 15

Figure S7 16

Figure S8 17

Figure S9 18

Figure S10 19

Supplementary Table S1 20

Supplementary Table S2 21

Supplementary Table S3 23

Supplementary Table S4 24

Reference 25

Experimental Section

Materials

Ni Sepharose^{TM} 6 fast flow was purchased from GE Healthcare. T4 ligase and the restriction enzyme BsaI were purchased from New England Biolabs. *E. coli* NEB 10-beta and *E. coli* BL21-AI (New England Biolabs) strains were used as host for cloning and protein expression. Unless otherwise indicated, all other chemicals were ordered from Sigma-Aldrich.

Computational modelling of PpSB1-LOV variants

For the introduction of the D-GAIQ-IALVF-ST-G-A-ST motif, first the C-terminal anchor residue was identified. This was done rationally by searching for residues whose side chain appeared close enough to the phosphate of the FMN to introduce a covalent bond to the new anchor residue. All visual inspection and molecular modeling were done with Yasara.[1] After modeling the covalent bond between anchor residue and FMN, additional mutations at the preceding six positions of the motif were introduced stepwise, in C- to N-terminal direction. The rational considerations used to select particular residues at a position are described in the results section. After introduction of the anchor residue, or any other new side chain, its ideal conformation (*i.e.* with least clashes and best interactions) was obtained using a rotamer search.[2] The commands used within Yasara for these tasks were "SwapRes" for mutagenesis and "OptimizeRes <name residue>, method = scwall" for the rotamer optimization.

To model the covalent attachment of the threonine to the FMN, a bond was created between the O_g of the introduced threonine and the phosphor atom of the FMN. This was done using Yasara's "AddBond" command after alchemically removing one oxygen of the phosphate and one hydrogen of the threonine using the "DelAtom" command. Subsequently, an energy minimization ("Experiment Minimization") was carried out using the NOVA force field, which is specifically optimized for structurally accurate modeling of proteins *in vacuo*.[3] During energy minimization, the tail of the FAD and the threonine side chain were allowed to move while the isoalloxazine ring and the rest of the protein were frozen ("FixAtom" command). The energy minimization started from very strained conformations and was observed to feature strong movements at the chiral C_b of the threonine. To correct for potential inversions of stereochemistry at that center, the threonine was re-introduced and the rotamer optimization, covalent bond formation, and energy minimization were repeated, but now starting from a far less strained conformation that could not result in chiral inversions.

To pre-screen the resulting variants for functionality, MD simulations and orthogonal three-dimensional structure predictions were carried out. MD simulations were performed and analyzed using established FRESCO protocols with all atoms free to move without any constraint.[4,5] FRESCO is a computational procedure to generate mutants with increased thermostability and it evaluates every mutant also using a multi-seeded MD simulation in explicit water. For each variant, five independently started MD simulations of a 100 ps were performed and the results were analyzed by visual inspection of the averaged structure over the last 50 ps of each of these trajectories. This allows to evaluate mutants for increased flexibility or structural defects that could be destabilizing. The protocols, including a detailed description on how to visually inspect for increased flexibility and find various structural problems, are published elsewhere.[4,5] All required Yasara simulation and inspection scripts are available via https://groups.google.com/g/fresco-stabilization-of-proteins. The three-dimensional structures of the mutants were also modeled orthogonally with AlphaFold2 using the CASP14 settings.[6] The miniSOGO variants was designed based on the similar way as PpSB1-LOV variants.

Plasmid construction

Codon-optimized versions of the genes encoding PpSB1-LOV from *Pseudomonas putida* KT2440, OYE from *Thermus scotoductus* and miniSOG from *Arabidopsis thaliana* were synthesized by Twist Bioscience. For the genes encoding BtNR variants with flavinylation recognition site were amplified by using PCR and the primers were list in **Supplementary Table 1**. All the genes were cloned into a pBAD-NHis6x-SUMO vector using the Golden Gate method. The Golden Gate products were transformed into *E. coli* NEB 10-beta. The plasmids were isolated and sent for sequencing (GATC, Germany) to confirm the correct ligation of the genes. SUMO-tagged wt and the variants of PpSB1-LOV, miniSOG, and BtNR were expressed by using the vector pBAD-NHis-SUMO in *E. coli* BL21-AI cells. The flavin transferase from *V. cholerae* (ApbE) and FAD synthetase from *C. ammoniagenes* (CaFADS) were cloned into multiple cloning site 1 (MCS-1; HindIII and NcoI) and MCS-2 (NdeI and PacI) of the pRSF-Duet-1 vector, respectively. All the protein sequence are shown in **Supplementary Table 2**.

Protein purification and characterization

The recombinant strain *E. coli* BL21-AI was used for expression of the PpSB1-LOV variants. For protein expression, transformed cells were incubated at 30° C for 20 h in 200 mL TB medium containing 50 μg/mL ampicillin. L-arabinose was added (0.02% w/v) when the OD600

was between 0.6 and 1.0. Cells were harvested by centrifugation (6,000 rpm, 20 minutes, 4ºC, Beckman-Coulter centrifuge), and the pellet was resuspended in 50 mM Tris-HCl, pH 8.0, containing 100 mM $MgCl_2$, 1.0 µg/mL DNase. Cells were lysed by sonication, followed by centrifugation (12,000 rpm, 30 minutes, 4°C). The N-His6x-SUMO-tagged proteins were purified using 4 mL HisTrap Ni-Sepharose HP columns (GE Healthcare Lifesciences, USA) and desalted with a HiPrep 26/10 desalting column (GE Healthcare Lifesciences), using 50 mM Tris-HCl buffer, pH 8.0. Protein aliquots were frozen using liquid nitrogen and stored at -70°C until further use. SUMO was cleaved from SUMO-tagged proteins by adding SUMO protease at 4°C overnight. Then SUMO free samples were used for gel filtration analysis on an ÄKTA purifier (GE Healthcare Lifesciences, USA) and ESI-MS analysis. Samples were applied to a Superdex 75 increase 10/300 GL column (GE Healthcare Lifesciences, USA). The column was equilibrated with 50 mM Tris-HCl (pH 8.0) buffer containing 200 mM NaCl.

For the coexpression of ApbE and CaFADS with all studied flavoproteins, *E. coli* BL21-AI cells were transformed with the modified pRSF-Duet-1 plasmid for expression of the flavin transferase and FAD synthetase together with the pBAD-based plasmid for expression of the respective flavoprotein variant. The cells were grown in 200 mL TB medium containing 50 µg/mL kanamycin and ampicillin. The expression was induced by adding 0.5 mM IPTG and 0.02 % L-arabinose at an OD_{600} of 0.8-1.0. Expression continued at 30 °C for 20 hours. Cells were harvested by centrifugation and purified as described above. Total protein concentration was determined by Bradford assay. The concentration of flavoproteins was determined by using the respective molar absorption coefficient (**Supplementary Table 3**). The molar absorption coefficients were determined by 0.2 % SDS treatment and were calculated from the absorption at 450 nm.

Absorption and fluorescence emission spectra were recorded with a V-650 UV-VIS spectrophotometer (JASCO) and a JASCO FP-8300 spectrofluorometer, respectively. Measurements were performed without previous illumination of the proteins in order to keep the proteins in the fluorescent on-state.

Determining non-covalently bound FMN

To measure the non-covalent binding FMN percentage in PpSB1-LOV-F1, the protein sample was denatured and precipitated with the TCA method as described in previous studies.[7] Briefly, TCA was added to the purified protein to a final concentration of 5% and then kept in ice for 1h, followed by centrifugation. The supernatant was collected and neutralized by the addition

of 1 M potassium phosphate (pH 7.0). The absorption spectra of the supernatant were recorded and used to calculate the FMN concentration by using the extinction coefficient of 12,200 M^{-1} cm^{-1} at 446 nm.

Thermal stability determination

To determine the thermostability of the studied flavoproteins, the apparent melting temperatures were measured by using the ThermoFluor assay method.[8] The samples (20 µL) contained 30 µM purified protein in 50 mM Tris-HCl, pH 8.0 and 1x SYPRO orange. Using an RT-PCR thermocycler, intensity of fluorescence was measured while the samples were heated up from 25 to 90°C with 0.5°C per step, using a holding time of 10 s at each step. The maximum of the first derivative of the observed flavin fluorescence was taken as the apparent melting temperature.

ESI-MS analysis of modified proteins

Electrospray ionization mass spectrometry (ESI-MS) was used to verify successful covalent incorporation of FMN. SUMO free samples were applied to ESI-MS using the Waters® Xevo® G2 Tof/ ACQUITY UPLC H-Class® System coupled to a quadrupole/time-of-flight (QToF) mass spectrometer equipped with a PDA detector. The eluent system employed was a combination of A (0.1% formic acid in water) and B (0.1% formic acid in acetonitrile) at a flow rate of 0.3 mL/min. Protein samples were separated on an Acquity BEH C4; 150 × 2.1 mm, 1.7 µm (Waters) column operated at 40°C. The sample injection volume was 4 µL. Mass spectra were obtained in the ESI-positive ion mode. Protein samples were diluted to 5 µM prior to analysis. Obtained charge density spectra were deconvoluted using the MagTran software. Protein was eluted using the following procedure: 0-2 min, 95 % A (5 % B); 2-15 min, gradient 95 to 5% A.

Fluorescence, photobleaching and photorecovery measurements

All spectroscopic analysis were carried out under dim light. Measurement of light-dependent absorption changes in the UV/Vis region (200–700 nm) was carried out using a V-650 UV-VIS spectrophotometer (JASCO). All measurements were performed at 25 °C. In order to generate the light state, samples were illuminated for 30 s using a blue light (435-445 nm spectral range) emitting LED lamp (Sigma-Aldrich, Micro Photochemical Reactor). The dark state recovery was measured from illuminated samples by recording the absorption recovery at 480 nm until

the baseline was reached. Recovery rate constants were determined by fitting to a single-exponential function.

For quantum yield (Φ_f) measurements, PpSB1-LOV variants and fluorescein (as a reference standard) were diluted in 1x Phosphate-buffered saline pH 7.4 ((137 mM NaCl, 2.7 mM KCl, 10 mM Na_2HPO_4, 1.8 mM KH_2PO_4) and 0.1 M NaOH buffer, respectively. Fluorescence spectra of individual samples were recorded by exciting the sample at 447 nm using a JASCO FP-8300 spectrofluorometer and measuring the fluorescence emission in the range of 460-700 nm. The fluorescence quantum yield was calculated as performed in a previous study.[9,10] Fluorescein (Φ_f^R = 0.91) was selected as reference.

Singlet oxygen detection

The Singlet Oxygen Sensor Green (SOSG) reagent was purchased from Thermo Fischer. The SOSG stock solutions were freshly prepared in methanol: the content of one vial (100 µg) was dissolved in 330 µL methanol to make a stock solution of 0.50 mM. The working solutions of the reagent were prepared immediately before use. In a reaction, the final concentrations were 1.0 µM SOSG, 10 µM miniSOG, 20 µM azide in SOSG buffer. The reaction mixture was illuminated using a blue light (435-445 nm spectral range) emitting LED lamp (Sigma-Aldrich, Micro Photochemical Reactor) for 15 min. The temperature was held constant at 25 °C. Fluorescence (excitation at 488 nm; emission at 525 nm) was recorded for 40 min in the microplatereader.

Enzyme kinetics of BtNR

To establish whether the flavinylated mutants BtNR F1-3 retained nitroreductase activity, we carried out steady-state kinetics experiments using nitrofurazone as substrate and NADPH as cofactor (**Supplementary Fig. 7**). The reactions were performed in potassium phosphate buffer pH 7.0, at 25°C. Because of its low solubility in water, nitrofurazone was dissolved in DMSO 4% (v/v). k_{cat} and K_m values for nitrofurazone were determined by following substrate depletion at 420 nm (ε420 = 5.59 M^{-1} cm^{-1}) using nitrofurazone at different concentrations (0-400 µM) and a fixed concentration of NADPH (100 µM for variant F1-F2 and 250 µM for variant F3). In all cases, reaction was initiated by the addition of the enzyme (final concentration up to 30 nM). Rates at different substrate concentrations were processed in GraphPad Prism 8.0 and fitted using a regular Michaelis-Menten formula resulting in apparent K_m (micromolar) and

apparent k_{cat} (inverse seconds) values. K_m and k_{cat} values of the flavinylated mutants for nitrofurazone were then compared with the values of BtNR wild-type.

OYE activity measurement

The activity measurement of OYE and its variants toward model substrate p-benzoquinone were determined by monitoring the consumption of NADPH at 340 nm ($\varepsilon = 6.22\ mM^{-1}\ cm^{-1}$). The reaction (100 µL) was prepared with 50 mM Tris-HCl buffer (pH = 7.5), 0.1 mM NADPH, and 1 mM p-benzoquinone. The reaction was initiated by adding the enzyme to the 50 nM final concentration.

Crystallization and structural determination

SUMO was cleaved from protein variants PpSB1-LOV-F1(R66T), MiniSOG-F2 (R57T) and BtNR-F3 (A17T) and samples were further purified by gel filtration chromatography at 280 K using a Superdex75 10/300 GL column (Cytiva), equilibrated with 20 mM HEPES buffer, pH 7.3, containing 150 mM NaCl. Fractions containing the protein of interest were pooled and concentrated to 33 mg mL^{-1} for PpSB1-LOV-F1, 11 mg mL^{-1} for MiniSOG-F2 and 10 mg mL^{-1} for BtNR-F3 using an Amicon Ultra centrifugal filter unit (Merck Millipore Ltd., USA) with an appropriate cut-off. Initial sitting-drop crystallization screening was performed using a Mosquito crystallization robot (STP Labtech) in 96-well MRC2 plates. Several commercially available crystallization screens were tested at 294K. Crystallization conditions were further optimized with the Dragonfly (STP Labtech). Crystals of PpSB1-LOV-F1 could be grown from PEG/Ion condition H7; 15% PEG3350, 10 mM $MgCl_2$, 5 mM $NiCl_2$ and 100 mM HEPES buffer pH 7.0. The addition of $NiCl_2$ to the experiment was essential for crystal growth. MiniSOG-F2 could be crystallized from condition G2 from JCSG+, and the optimized condition contains 19% polyacrylic acid 5100 and 100 mM HEPES buffer pH 7.5. Crystals of BtNR-F3 were grown from PACT conditions C3-C5, 25% PEG1500 and PCB buffer pH 6-8. None of the crystals of the proteins could be grown form previous reported crystallization conditions of their wild-type (or mutated) proteins.

Prior to data collection, crystals were briefly soaked in a cryoprotectant solution containing the crystallization solution supported with 25% glycerol, and flash-cooled in liquid nitrogen. X-ray diffraction data were collected at ESRF beamline MASSIF-1[11] . Intensity data were integrated with XDS [12] and scaled with the AIMLESS routine [13] from the CCP4 software suite[14]. Molecular replacement was performed with PHASER [15], using PDB code 3SW1 [16] for PpSB1-

LOV-F1, 6GPU for MiniSOG [17] and wild-type BtNR (unpublished) as search models. The resulting structures were improved by several rounds of model building and refinement, using the programs Coot [18] and REFMAC5[19], alternately. The cofactor FMN was covalently attached to the proteins. The quality of the models was analyzed with PDB_REDO[20] and MolProbity [21]. PyMOL (Schrödinger) was used for structure analysis and figure preparation. Data collection statistics and refinement details are listed in **Supplementary Table S4**.

Supplementary Figures and Tables

Figure S1

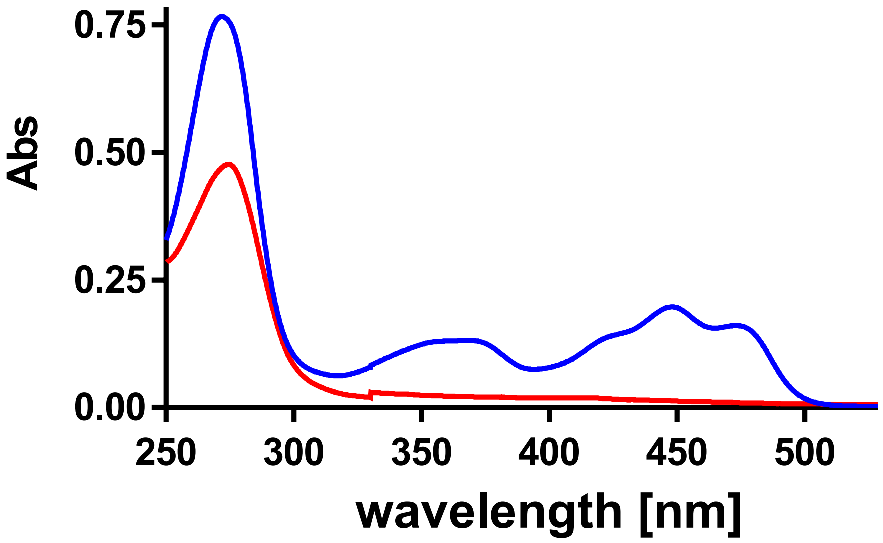

Supplementary Figure S1. Absorption spectra of purified PpSB1-LOV F2, with and without coexpression of ApbE (blue and red lines, respectively).

Figure S2

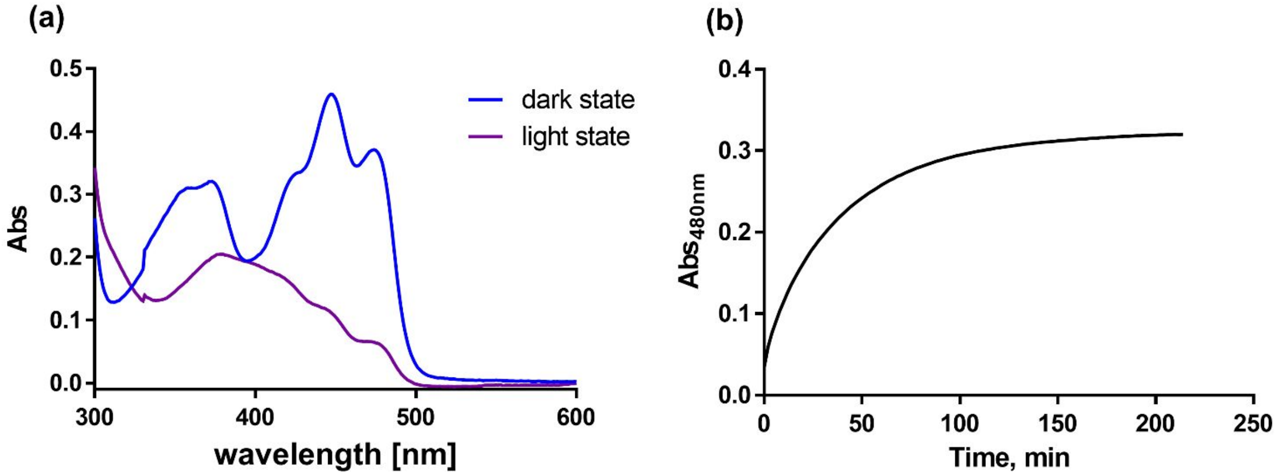

Supplementary Figure S2. Photochemical property of PpSB1-LOV F1a expressed in absence of ApbE. (A) The absorption spectrum of PpSB1-LOV F1a, harboring non-covalent FMN, in the dark state (blue line) and in the light state (purple line). (B) Dark recovery as measured by recovery of absorbance at 480 nm.

Figure S3

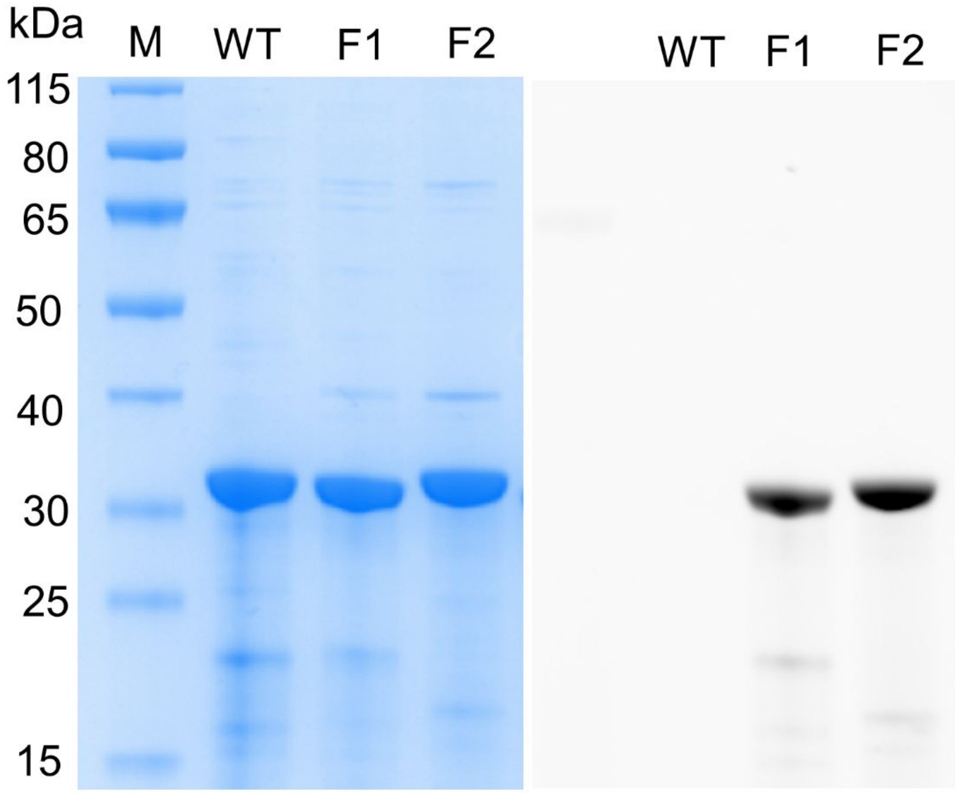

Supplementary Figure S3. Full images for gel data presented in the Figure 2. Left: Polyacrylamide gel (SDS-PAGE) of purified PpSB1-LOV proteins: wild-type, variant F1, and variant F2. Right: In-gel fluorescence (right) and protein-staining (left) of the same gel is shown.

Figure S4

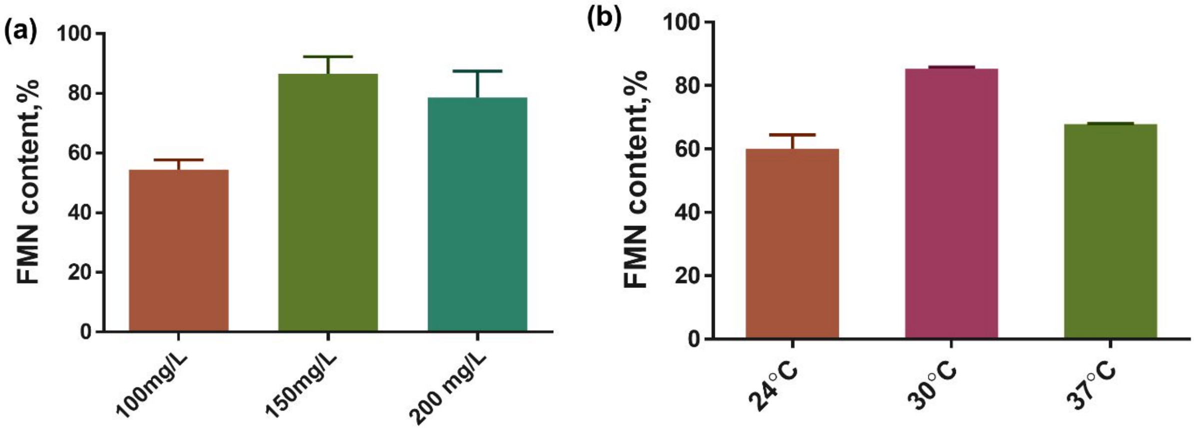

Supplementary Figure S4. (A) Effect of riboflavin concentration in the culture medium on covalent flavinylation of PpSB1-LOV-F1 at 30 ºC. (B) Effect of expression temperature on covalent flavinylation of PpSB1-LOV-F1 while adding 150 mg/L riboflavin.

Figure S5

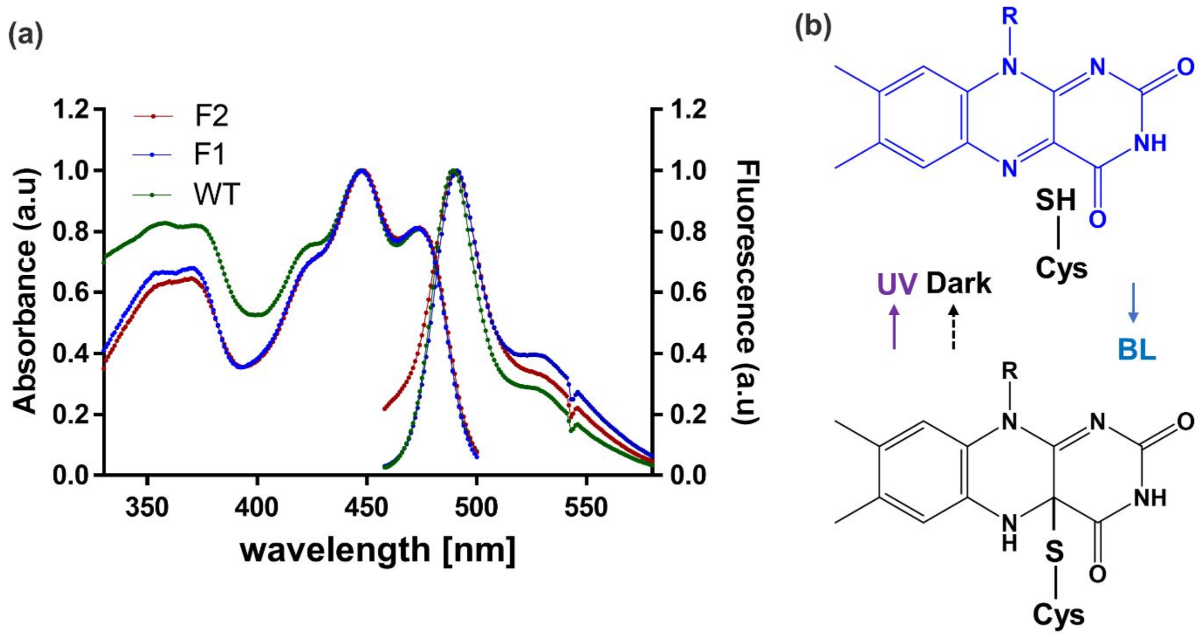

Supplementary Figure S5: Spectral properties and photocycle of the PpSB1-LOV protein. (A) Absorption and emission spectra of PpSB1-LOV variants. The fluorescence emission spectra were measured using an excitation wavelength of 447 nm. (B) Mechanism of photosensing of PpSB1-LOV: blue light (BL) triggers formation of a cysteinyl-C4a covalent adduct which reverts to the resting state in dark or by UV illumination.

Figure S6

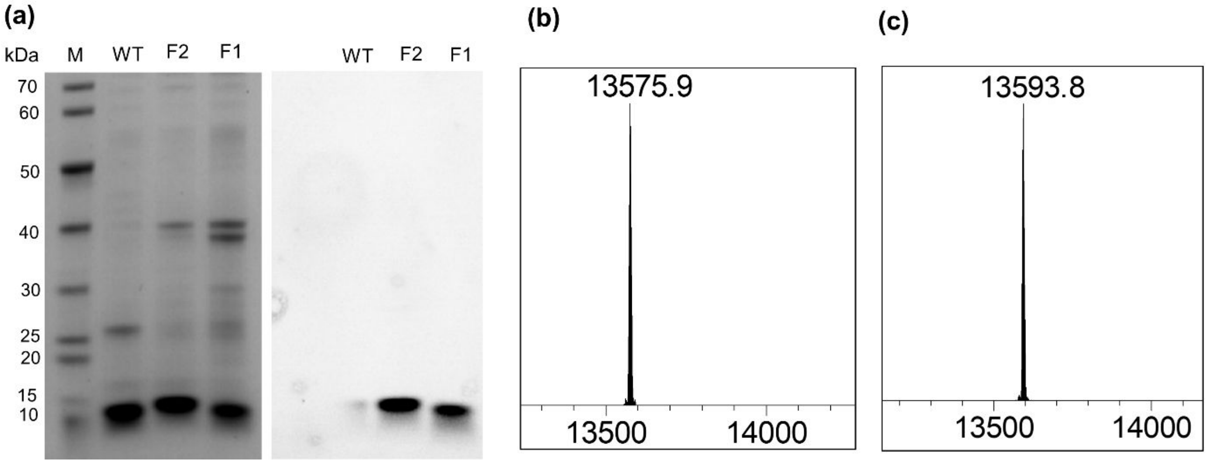

Supplementary Figure S6. Expression and mass identification of mini-SOG its variants. (A) Polyacrylamide gel (SDS-PAGE) of purified mini-SOG: wild-type, variant F1, variant F2. In-gel fluorescence (right) and protein-staining (left) of the same gel is shown. Electrospray ionization mass spectral analysis of F1 (B), and F2 (C).

Figure S7

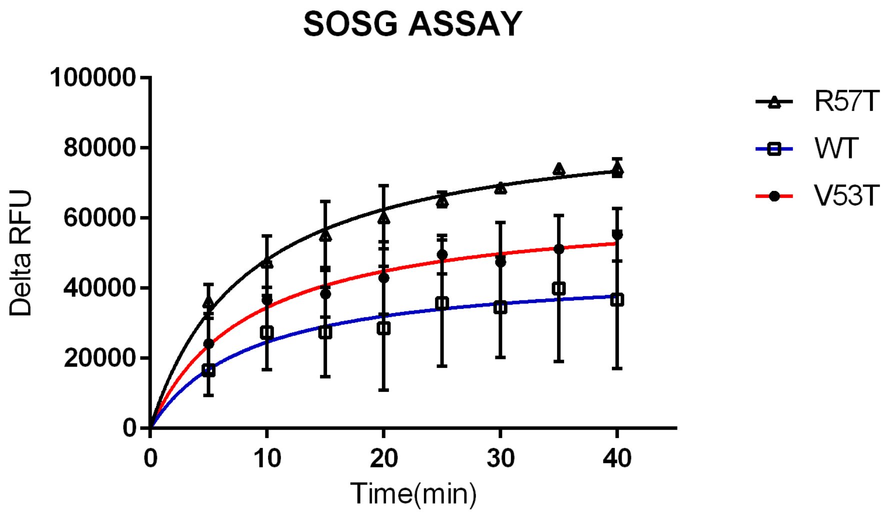

Supplementary Figure S7. Detection of singlet oxygen generated by miniSOG variants. The fluorescence intensity reflects the amount of formed singlet oxygen ($^1O_2$).

Figure S8

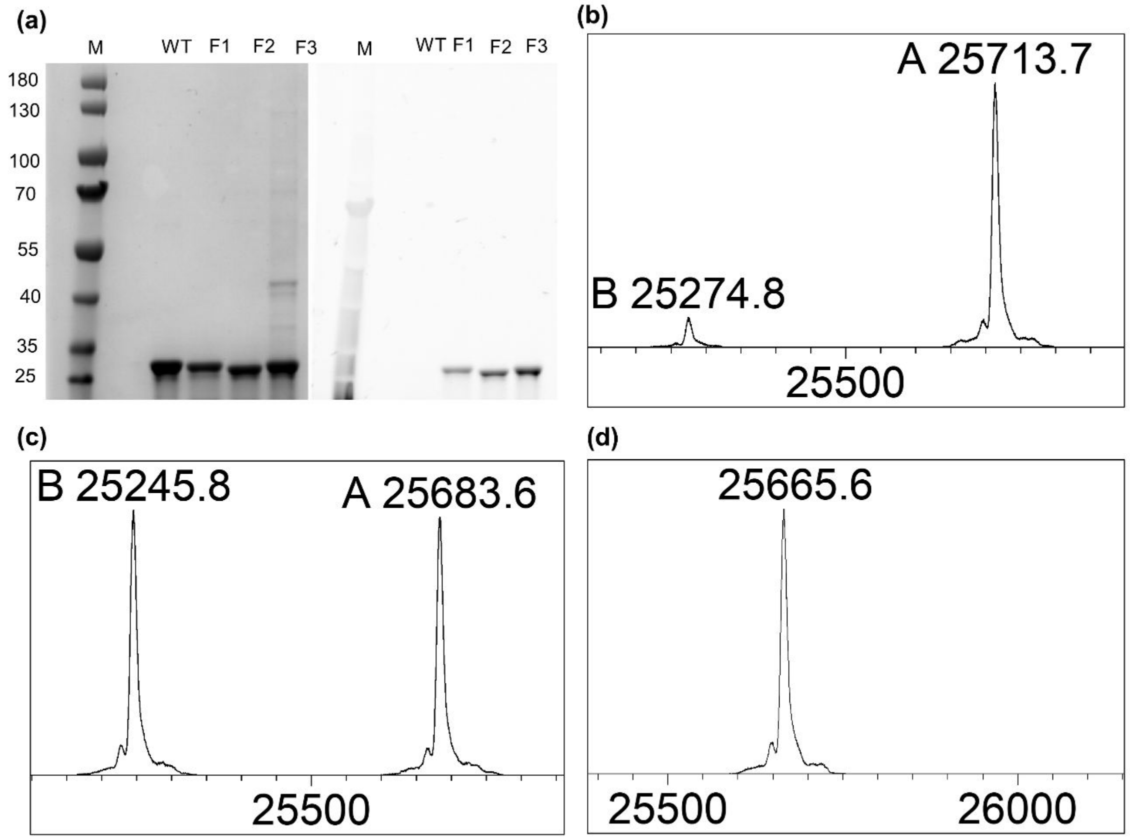

Supplementary Figure S8. Expression and mass identification of BtNR and its variants. (A) Polyacrylamide gel (SDS-PAGE) of purified SUMO-tag free BtNR: wild-type, variant F1, variant F2, and variant F3. In-gel fluorescence (right) and protein-staining (left). Electrospray ionization mass spectral analysis of F1 (B), F2 (C), and F3 (D).

Figure S9.

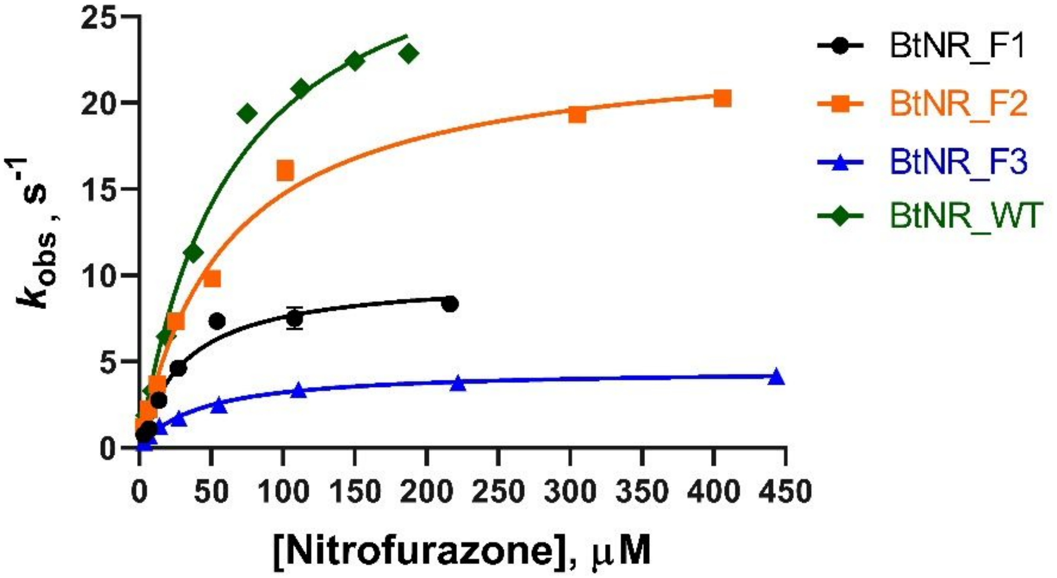

Supplementary Figure S9. Steady-state kinetics of BtNR wild-type and flavinylated mutants F1-3. Steady-state kinetics of nitrofurazone in the presence of 100 μM NADPH (WT, F1, F2) or 250 μM NADPH (F3). The reaction was initiated by adding 20-30 nM purified enzyme. All reactions were performed in duplicates in 100 mM potassium phosphate buffer, pH 7.0 at 25°C.

Figure S10.

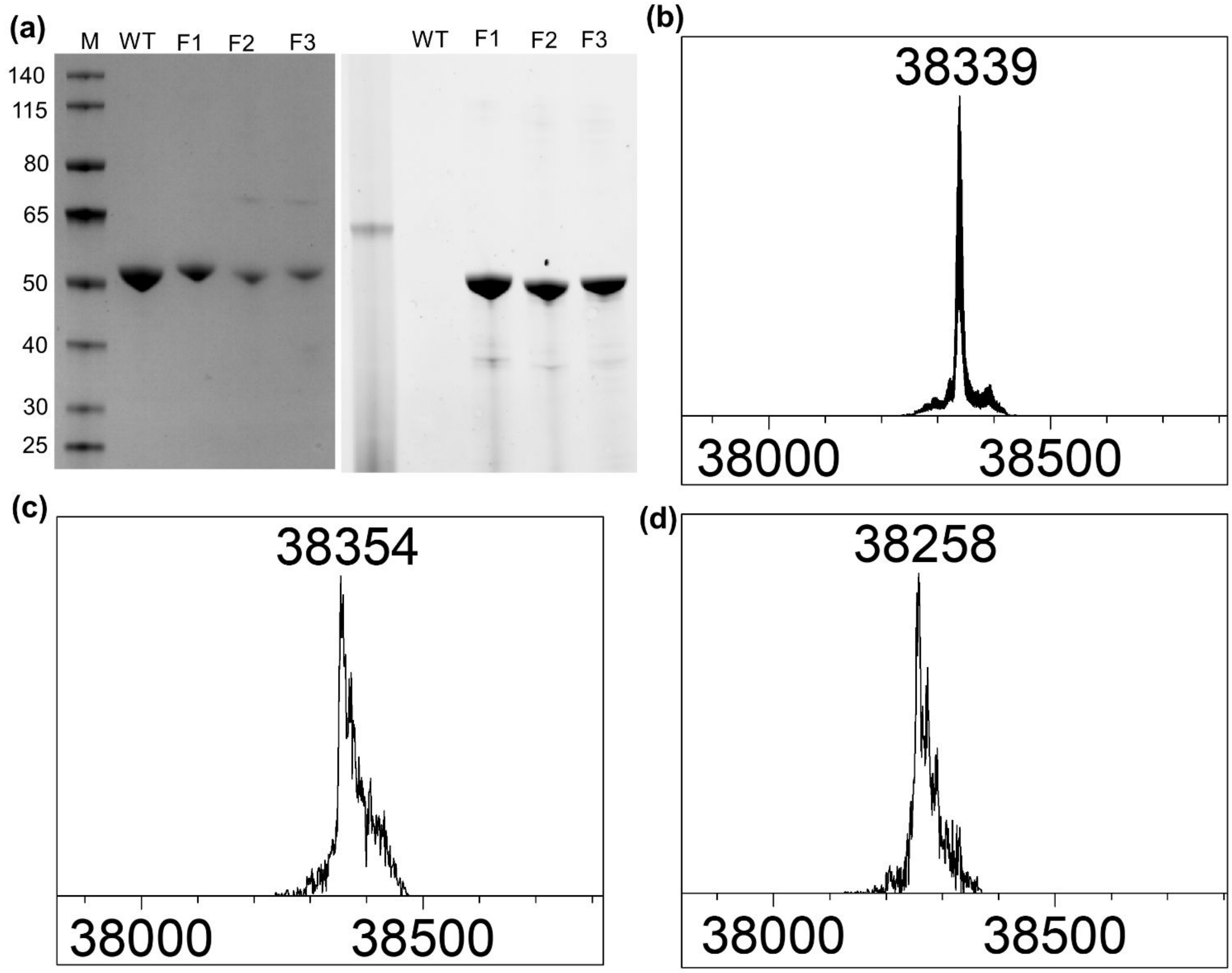

Supplementary Figure S10. Expression and mass identification of OYE (3HGJ) and its variants. (A) Polyacrylamide gel (SDS-PAGE) of purified OYEs: wild-type, variant F1, variant F2, and variant F3. In-gel fluorescence (right) and protein-staining (left) of the same gel is shown. Electrospray ionization mass spectral analysis of F1 (B), F2 (C), and F3 (D).

Supplementary Table S1.

All the primers that used for cloning BtNR variants in this study.

Protein	Protein sequence
BtNR-F1-FW	GAGGTCTCGTGGTATGGCTGACGTTAAAAAACAGATCGATGCGGCTAGCGGCGCCACCCACGCTACCAAAGAATTCGAC
BtNR-F2-FW	GAGGTCTCGTGGTATGGCTGACGTTAAAAAACAGATCCTGGACGCTCTGAGCGGCGCCACCGCTACCAAAGAATTCGACCCGAACA
BtNR-F3-FW	GAGGTCTCGTGGTATGGCTGACGTTAAAAAACAGATCCTGGACGATGGCCTGAGCGGCGCCACCACCAAAGAATTCGACCCGAA
BtNR-WT-FW	GAGGTCTCGTGGTATGGCTGACGTTAAAAAAC
BtNR-RV	GTGGTCTCGCAAGTTAAACCCATTTAACAACG

The introduced BsaI sites are shown in red.

Supplementary Table S2.

Sequences of proteins used in this study.

Protein	Protein sequence
PpSB1-LOV<br>*(Pseudomonas putida,* 5J3W*)*	MINAQLLQSMVDASNDGIVVAEKEGDDTILIYVNAAFEYLTGYSRDEILY<br>QDCRFLQGDDRDQLGRARIRKAMAEGRPCREVLRNYRKDGSAFWNELSI<br>TPVKSDFDQRTYFIGIQKDVSRQVELERELAELRARPKPDERA
PpSB1-LOV-F1	MINAQLLQSMVDASNDGIVVAEKEGDDTILIYVNAAFEYLTGYSRDEILY<br>QDCRFLQGDDAASGATARIRKAMAEGRPCREVLRNYRKDGSAFWNELSI<br>TPVKSDFDQRTYFIGIQKDVSRQVELERELAELRARPKPDERA
PpSB1-LOV-F2	MINAQLLQSMVDASNDGIVVAEKEGDDTILIYVNAAFEYLTGYSRDEILY<br>QDCRFLQGDDRDQDGASGATKAMAEGRPCREVLRNYRKDGSAFWNELS<br>ITPVKSDFDQRTYFIGIQKDVSRQVELERELAELRARPKPDERA
miniSOG<br>*(Arabidopsis thaliana,* 6GPU)	MEKSFVITDPRLPDNPIIFASDGFLELTEYSREEILGRNGRFLQGPETDQAT<br>VQKIRDAIRDQREITVQLINYTKSGKKFWNLLHLQPMRDQKGELQYFIGV<br>QLDGEFIPNPLLG
miniSOG-F1	MEKSFVITDPRLPDNPIIFASDGFLELTEYSREEILGRNGRFLQGPDAASGA<br>TQKIRDAIRDQREITVQLINYTKSGKKFWNLLHLQPMRDQKGELQYFIGV<br>QLDGEFIPNPLLG
miniSOG-F2	MEKSFVITDPRLPDNPIIFASDGFLELTEYSREEILGRNGRFLQGPETDQDI<br>VSGATDAIRDQREITVQLINYTKSGKKFWNLLHLQPMRDQKGELQYFIGV<br>QLDGEFIPNPLLG
BtNR<br>(*Bacillus tequilensis* nitroreductase)	MADVKKQILDAYNFRHATKEFDPNKKVSDSDFEFILETGRLSPSSLGLEP<br>WKFVVVQNPEFREKLREYTWGAQKQLPTASHFVLILARTAKDIKYNADY<br>IKRHLKEVKQMPQDVSEGYISKTEEFQKNDLHLLESDRTLFDWASKQTYI<br>ALGNMMTAAAQIGVDSCPIEGFQYDHIHRILEEEGLLENGSFDISVMAAF<br>GYRVRDPRPKTRSAVEDVVKWV
BtNR-F1	MADVKKQIDAASGATHATKEFDPNKKVSDSDFEFILETGRLSPSSLGLEP<br>WKFVVVQNPEFREKLREYTWGAQKQLPTASHFVLILARTAKDIKYNADY<br>IKRHLKEVKQMPQDVSEGYISKTEEFQKNDLHLLESDRTLFDWASKQTYI<br>ALGNMMTAAAQIGVDSCPIEGFQYDHIHRILEEEGLLENGSFDISVMAAF<br>GYRVRDPRPKTRSAVEDVVKWV
BtNR-F2	MADVKKQILDALSGATATKEFDPNKKVSDSDFEFILETGRLSPSSLGLEP<br>WKFVVVQNPEFREKLREYTWGAQKQLPTASHFVLILARTAKDIKYNADY<br>IKRHLKEVKQMPQDVSEGYISKTEEFQKNDLHLLESDRTLFDWASKQTYI<br>ALGNMMTAAAQIGVDSCPIEGFQYDHIHRILEEEGLLENGSFDISVMAAF<br>GYRVRDPRPKTRSAVEDVVKWV
BtNR-F3	MADVKKQILDDGLSGATTKEFDPNKKVSDSDFEFILETGRLSPSSLGLEP<br>WKFVVVQNPEFREKLREYTWGAQKQLPTASHFVLILARTAKDIKYNADY<br>IKRHLKEVKQMPQDVSEGYISKTEEFQKNDLHLLESDRTLFDWASKQTYI<br>ALGNMMTAAAQIGVDSCPIEGFQYDHIHRILEEEGLLENGSFDISVMAAF<br>GYRVRDPRPKTRSAVEDVVKWV
Old yellow enzyme<br>(*Thermus scotoductus,* PDB:3HGJ)	MALLFTPLELGGLRLKNRLAMSPMCQYSATLEGEVTDWHLLHYPTRAL<br>GGVGLILVEATAVEPLGRISPYDLGIWSEDHLPGLKELARRIREAGAVPGI<br>QLAHAGRKAGTARPWEGGKPLGWRVVGPSPIPFDEGYPVPEPLDEAGME<br>RILQAFVEGARRALRAGFQVIELHMAHGYLLSSFLSPLSNQRTDAYGGSL<br>ENRMRFPLQVAQAVREVVPRELPLFVRVSATDWGEGGWSLEDTLAFAR<br>RLKELGVDLLDCSSGGVVLRVRIPLAPGFQVPFADAVRKRVGLRTGAVG<br>LITTPEQAETLLQAGSADLVLLGRVLLRDPYFPLRAAKALGVAPEVPPQY<br>QRGF
OYE-F1	MALLFTPLELGGLRLKNRLAMSPMCQYSATLEGEVTDWHLLHYPTRAL<br>GGVGLILVEATAVEPLGRISPYDLGIWSEDHLPGLKELARRIREAGAVPGI<br>QLAHAGRKAGTARPWEGGKPLGWRVVGPSPIPFDEGYPVPEPLDEAGME<br>RILQAFVEGARRALRAGFQVIELHMAHGYLLSSFLSPLSNQRTDAYGGSL

	ENRMRFPLQVAQAVREVVPRELPLFVRVSATDWGEGGWSLEDTLAFAR<br>RLKELGVDLLDCSSGGVVLRVRIPLAPGFQVPFADAVRKRVGLDAASGA<br>TITTPEQAETLLQAGSADLVLLGRVLLRDPYFPLRAAKALGVAPEVPPQY<br>QRGF
OYE-F2	MALLFTPLELGGLRLKNRLAMSPMCQYSATLEGEVTDWHLLHYPTRAL<br>GGVGLILVEATAVEPLGRISPYDLGIWSEDHLPGLKELARRIREAGAVPGI<br>QLAHAGRKAGTARPWEGGKPLGWRVVGPSPIPFDEGYPVPEPLDEAGME<br>RILQAFVEGARRALRAGFQVIELHMAHGYLLSSFLSPLSNQRTDAYGGSL<br>ENRMRFPLQVAQAVREVVPRELPLFVRVSATDWGEGGWSLEDTLAFAR<br>RLKELGVDLLDCSSGGVVLRVRIPLAPGFQVPFADAVRKRVGLRTGAVG<br>LITTPEQAETLLQAGSDALSGATRVLLRDPYFPLRAAKALGVAPEVPPQY<br>QRGF
OYE-F3	MALLFTPLELGGLRLKNRLAMSPMCQYSATLEGEVTDWHLLHYPTRAL<br>GGVGLILVEATAVEPLGRISPYDLGIWSEDHLPGLKELARRIREAGAVPGI<br>QLAHAGRKAGTARPWEGGKPLGWRVVGPSPIPFDEGYPVPEPLDEAGME<br>RILQAFVEGARRALRAGFQVIELHMAHGYLLSSFLSPLSNQRTDAYGGSL<br>ENRMRFPLQVAQAVREVVPRELPLFVRVSATDWGEGGWSLEDTLAFAR<br>RLKELGVDLLDCSSGGVVLRVRIPLAPGFQVPFADAVRKRVGLRTGAVG<br>LITTPEQAETLLQAGSADAVSGATVLLRDPYFPLRAAKALGVAPEVPPQY<br>QRGF
VcApbE	MEKPAEQVHLSGPTMGTTYNIKYIQQPGIADSKILQTEIDRLLEEVNDQM<br>STYRKDSELSRFNQHTSSEPFAVSTQTLTVVKEAIRLNGLTEGALDVTVGP<br>LVNLWGFGPEARPDVVPTDEELNARRAITGIEHLTIEGNTLSKDIPELYVD<br>LSTIAKGWGVDVVADYLQSQGIENYMVEIGGEIRLKGLNRDGVPWRIAIE<br>KPSVDQRSVQEIIEPGDYAIATSGDYRNYFEQDGVRYSHIIDPTTGRPINNR<br>VVSVTVLDKSCMTADGLATGLMVMGEERGMAVAEANQIPVLMIVKTD<br>DGFKEYASSSFKPFLSK
CaFADs	MDIWYGTAAVPKDLDNSAVTIGVFDGVHRGHQKLINATVEKAREVGAK<br>AIMVTFDPHPVSVFLPRRAPLGITTLAERFALAESFGIDGVLVIDFTRELSG<br>TSPEKYVEFLLEDTLHASHVVVGANFTFGENAAGTADSLRQICQSRLTVD<br>VIDLLDDEGVRISSTTVREFLSEGDVARANWALGRHFYVTGPVVRGAGR<br>GGKELGFPTANQYFHDTVALPADGVYAGWLTILPTEAPVSGNMEPEVAY<br>AAAISVGTNPTFGDEQRSVESFVLDRDADLYGHDVKVEFVDHVRAMEKF<br>DSVEQLLEVMAKDVQKTRTLLAQDVQAHKMAPETYFLQAES

The introduced covalent flavinylation motifs are shown in red.

Supplementary Table S3.

Molar extinction coefficients of PpSB1-LOV, MiniSOG, and BtNR proteins.

**protein**	**ε [$M^{-1}\cdot cm^{-1}$]**
PpSB1-LOV -WT	13,200
PpSB1-LOV-F1	13,400
PpSB1-LOV -F2	13,900
MiniSOG-WT	16,350
MiniSOG-F1	20,800
MiniSOG-F2	21,950
BtNR-WT	11,371
BtNR-F1	12,500
BtNR-F2	12,840
BtNR-F3	9,900

Supplementary Table S4.

Data collection and refinement statistics.

Data collection	PpSB1-LOV-F1	MiniSOG-F2	BtNR-F3
Resolution range (Å)	76.9 - 2.40 (2.49- 2.40)	67.4 -2.00 (2.05 - 2.00)	48.6 – 2.00 (2.05 - 2.00)
Space group	H3	$C222_1$	$P2_12_12_1$
Unit cell dimensions a,b,c, (Å)<br>Angles (°)	153.9, 153.8, 36.0<br>90, 90 ,120	62.8, 130.3, 67.4<br>90, 90, 90	48.6 62.6 135.8<br>90, 90, 90
V_M ($Å^3$/Da) / Solvent (%)	2.4 / 49	2.6 / 52	2.0 /39
Observed reflections	22,897 (2,476)	69,790 (5,601)	173,576 (13,430)
Unique reflections	11,764 (1,262)	17,411(1,375)	28,377 (2,089)
$CC_{(1/2)}$	0.968 (0.722)	0.999 (0.697)	0.885 (0.720)
$<I/\sigma>$	11.8 (1.6)	11.9 (1.6)	13.7 (2.9)
R_{merge}	0.035 (0.452)	0.062 (0.931)	0.201 (1.34)
R_{pim}	0.035(0.452)	0.036 (0.514)	0.107 (0.648)
Completeness (%)	94.7 (97.0)	91.8 (98.5)	98.5 (99.9)
Multiplicity	1.9 (2.0)	4.0 (4.1)	6.1 (6.4)
Refinement			
Atoms in A.U. protein / metal / FMN / waters	2156 / 1 / 60 / -	1830 /-/ 60 / 85	3403 / -/ 60 / 154
Average B-factors protein / FMN / solvent ($Å^2$)	74.1 / 72.0/-	37.6 / 31.8 / 43.3	23.3 / 34.7 / 29.6
R_{work} / R_{free} (%)	22.7 / 29.4	18.8 / 22.8	19.3 / 23.4
RMSD bond lengths (Å), bond angles	0.009, 1.7	0.008, 1.5	0.009, 1.5
Ramachandran preferred/outliers (%)	96.2 / 1.50	99.1 / 0.0	97.5 / 0.0
Rotamers preferred/outliers (%)	83.6 / 6.6	92.8 / 3.6	91.5 /3.3
Clash score (percentile)	10.99 (93rd)	5.28 (97th)	2.98 (99th)
Molprobity score (percentile)	2.18 (87th)	1.71 (90th)	1.48 (97th)
PDB accession code	8Q5E	8Q5F	8Q5G

Numbers in parenthesis are for the highest resolution shell.

Reference

(1) Krieger, E.; Vriend, G. YASARA View - Molecular Graphics for All Devices - from Smartphones to Workstations. *Bioinformatics* **2014**, *30* (20), 2981–2982. https://doi.org/10.1093/bioinformatics/btu426.

(2) Krieger, E.; Joo, K.; Lee, J.; Lee, J.; Raman, S.; Thompson, J.; Tyka, M.; Baker, D.; Karplus, K. Improving Physical Realism, Stereochemistry, and Side-Chain Accuracy in Homology Modeling: Four Approaches That Performed Well in CASP8. *Proteins: Structure, Function and Bioinformatics* **2009**, *77* (S 9), 114–122. https://doi.org/10.1002/prot.22570.

(3) Krieger, E.; Koraimann, G.; Vriend, G. Increasing the Precision of Comparative Models with YASARA NOVA - A Self-Parameterizing Force Field. *Proteins: Structure, Function and Genetics* **2002**, *47* (3), 393–402. https://doi.org/10.1002/prot.10104.

(4) Wijma, H. J.; Fürst, M. J. L. J.; Janssen, D. B. A Computational Library Design Protocol for Rapid Improvement of Protein Stability: FRESCO BT - Protein Engineering: Methods and Protocols. *Methods in Molecular Biology* **2018**, *1685*, 69–85. https://doi.org/10.1007/978-1-4939-7366-8_5.

(5) Wijma, H. J.; Floor, R. J.; Jekel, P. A.; Baker, D.; Marrink, S. J.; Janssen, D. B. Computationally Designed Libraries for Rapid Enzyme Stabilization. *Protein Engineering, Design and Selection* **2014**, *27* (2), 49–58. https://doi.org/10.1093/protein/gzt061.

(6) Jumper, J.; Evans, R.; Pritzel, A.; Green, T.; Figurnov, M.; Ronneberger, O.; Tunyasuvunakool, K.; Bates, R.; Žídek, A.; Potapenko, A.; Bridgland, A.; Meyer, C.; Kohl, S. A. A.; Ballard, A. J.; Cowie, A.; Romera-Paredes, B.; Nikolov, S.; Jain, R.; Adler, J.; Back, T.; Petersen, S.; Reiman, D.; Clancy, E.; Zielinski, M.; Steinegger, M.; Pacholska, M.; Berghammer, T.; Bodenstein, S.; Silver, D.; Vinyals, O.; Senior, A. W.; Kavukcuoglu, K.; Kohli, P.; Hassabis, D. Highly Accurate Protein Structure Prediction with AlphaFold. *Nature* **2021**, *596* (7873), 583–589. https://doi.org/10.1038/s41586-021-03819-2.

(7) Barquera, B.; Hellwig, P.; Zhou, W.; Morgan, J. E.; Häse, C. C.; Gosink, K. K.; Nilges, M.; Bruesehoff, P. J.; Roth, A.; Lancaster, C. R. D.; Gennis, R. B. Purification and Characterization of the Recombinant Na^+-Translocating NADH:Quinone Oxidoreductase from *Vibrio cholerae*. *Biochemistry* **2002**, *41* (11), 3781–3789. https://doi.org/10.1021/bi011873o.

(8) Lo, M. C.; Aulabaugh, A.; Jin, G.; Cowling, R.; Bard, J.; Malamas, M.; Ellestad, G. Evaluation of Fluorescence-Based Thermal Shift Assays for Hit Identification in Drug Discovery. *Analytical Biochemistry* **2004**, *332* (1), 153–159. https://doi.org/10.1016/j.ab.2004.04.031.

(9) Tong, Y.; Loonstra, M. R.; Fraaije, M. W. Broadening the Scope of the Flavin-Tag Method by Improving Flavin Incorporation and Incorporating Flavin Analogs. *ChemBioChem* **2022**, *23*, 1–8. https://doi.org/10.1002/cbic.202200144.

(10) Ko, S.; Ko, S.; Jeon, H.; Yoon, S.; Kyung, M.; Yun, H.; Na, J. H.; Jung, S. T. Discovery of Novel *Pseudomonas putida* Flavin-Binding Fluorescent Protein Variants with Significantly Improved Quantum Yield. *Journal of Agricultural and Food Chemistry* **2020**, *68* (21), 5873–5879. https://doi.org/10.1021/acs.jafc.0c00121.

(11) Bowler, M. W.; Nurizzo, D.; Barrett, R.; Beteva, A.; Bodin, M.; Caserotto, H.; Delagenière, S.; Dobias, F.; Flot, D.; Giraud, T.; Guichard, N.; Guijarro, M.; Lentini, M.; Leonard, G. A.; McSweeney, S.; Oskarsson, M.; Schmidt, W.; Snigirev, A.; Von Stetten, D.; Surr, J.; Svensson, O.; Theveneau, P.; Mueller-Dieckmann, C. MASSIF-1: A Beamline Dedicated to the Fully Automatic Characterization and Data Collection from Crystals of Biological Macromolecules. *Journal of Synchrotron Radiation* **2015**, *22*, 1540–1547. https://doi.org/10.1107/S1600577515016604.

(12) Kabsch, W. XDS. *Acta Crystallogr D Biol Crystallogr* **2010**, *66* (2), 125–132. https://doi.org/10.1107/S0907444909047337.

(13) Evans, P. Scaling and Assessment of Data Quality. In *Acta Crystallographica Section D: Biological Crystallography*; 2006; Vol. 62, pp 72–82. https://doi.org/10.1107/S0907444905036693.

(14) Winn, M. D.; Ballard, C. C.; Cowtan, K. D.; Dodson, E. J.; Emsley, P.; Evans, P. R.; Keegan, R. M.; Krissinel, E. B.; Leslie, A. G. W.; McCoy, A.; McNicholas, S. J.; Murshudov, G. N.; Pannu, N. S.; Potterton, E. A.; Powell, H. R.; Read, R. J.; Vagin, A.; Wilson, K. S. Overview of the CCP4 Suite and Current Developments. *Acta Crystallographica Section D: Biological Crystallography*. April 2011, pp 235–242. https://doi.org/10.1107/S0907444910045749.

(15) McCoy, A. J.; Grosse-Kunstleve, R. W.; Adams, P. D.; Winn, M. D.; Storoni, L. C.; Read, R. J. Phaser Crystallographic Software. *Journal Applied Crystallogr* **2007**, *40* (4), 658–674. https://doi.org/10.1107/S0021889807021206.

(16) Circolone, F.; Granzin, J.; Jentzsch, K.; Drepper, T.; Jaeger, K. E.; Willbold, D.; Krauss, U.; Batra-Safferling, R. Structural Basis for the Slow Dark Recovery of a Full-Length LOV Protein from *Pseudomonas putida*. *Journal of Molecular Biology* **2012**, *417* (4), 362–374. https://doi.org/10.1016/j.jmb.2012.01.056.

(17) Torra, J.; Lafaye, C.; Signor, L.; Aumonier, S.; Flors, C.; Shu, X.; Nonell, S.; Gotthard, G.; Royant, A. Tailing MiniSOG: Structural Bases of the Complex Photophysics of a Flavin-Binding Singlet Oxygen Photosensitizing Protein. *Scientific Reports* **2019**, *9* (1). https://doi.org/10.1038/s41598-019-38955-3.

(18) Emsley, P.; Lohkamp, B.; Scott, W. G.; Cowtan, K. Features and Development of Coot. *Acta Crystallogr D Biol Crystallogr* **2010**, *66* (4), 486–501. https://doi.org/10.1107/S0907444910007493.

(19) Murshudov, G. N.; Skubák, P.; Lebedev, A. A.; Pannu, N. S.; Steiner, R. A.; Nicholls, R. A.; Winn, M. D.; Long, F.; Vagin, A. A. REFMAC5 for the Refinement of Macromolecular Crystal Structures. *Acta Crystallogr D Biol Crystallogr* **2011**, *67* (4), 355–367. https://doi.org/10.1107/S0907444911001314.

(20) Joosten, R. P.; Long, F.; Murshudov, G. N.; Perrakis, A. The PDB_REDO Server for Macromolecular Structure Model Optimization. *IUCrJ* **2014**, *1*, 213–220. https://doi.org/10.1107/S2052252514009324.

(21) Chen, V. B.; Arendall, W. B.; Headd, J. J.; Keedy, D. A.; Immormino, R. M.; Kapral, G. J.; Murray, L. W.; Richardson, J. S.; Richardson, D. C. MolProbity: All-Atom Structure Validation for Macromolecular Crystallography. *Acta Crystallogr D Biol Crystallogr* **2010**, *66* (1), 12–21. https://doi.org/10.1107/S0907444909042073.
